# Temporal and spatial comparisons of the reproductive biology of northern Gulf of Mexico (USA) red snapper (*Lutjanus campechanus*) collected a decade apart

**DOI:** 10.1371/journal.pone.0172360

**Published:** 2017-03-29

**Authors:** Dannielle H. Kulaw, James H. Cowan, Melissa W. Jackson

**Affiliations:** 1 Department of Oceanography and Coastal Sciences, Louisiana State University, Baton Rouge, Louisiana, United States of America; 2 Department of Marine Sciences, University of South Alabama, Mobile, Alabama, United States of America; Aristotle University of Thessaloniki, GREECE

## Abstract

In studies done a decade apart, we provide evidence of a recent shift toward a slower progression to sexual maturity as well as reduced egg production, especially among young, small female red snapper, in the Gulf of Mexico (Gulf). Slower maturation rates (among fish ≤6 years old), lower GSI values and decreased spawning frequency were observed, and were especially pronounced in the northwestern Gulf. Furthermore, an Index of Reproductive Importance showed that young fish (ages 2–7) are contributing far less to the spawning stock in recent years, while older fish (>8 years) are contributing more, when compared to fish from the same age groups sampled in the previous decade. Coincident with these changes in reproductive output, fishing pressure has steadily declined gulf-wide, and spawning stock biomass and spawning potential ratio have increased. Thus, it is possible that the age structure of the red snapper stock is becoming less truncated, or that reproductive effort observed is due to the temporary influence of recent strong year classes produced in 2004 and 2006 as they begin to reach full reproductive potential. If the latter is true, careful documentation of the stock’s reproductive dynamics during a time of population growth provides new understanding at the meta-population spatial and decadal temporal scales. In contrast, if the former is true, a truncated age structure due to overharvest can limit the productivity of the Gulf red snapper stock. In addition, we have learned that red snapper females in the northwestern Gulf collected on natural reefs and banks have much higher reproductive output than those on artificial reefs in the form of standing and toppled oil and gas platforms, thus making the need to know the relative abundance of females found on these disparate habitats an important next step toward better-understanding factors impacting the reproductive dynamics of this species.

## Introduction

‘While an improved understanding of spatial differences in life history and demographics is much needed, it is no less important to understand the degree to which life history and demographic attributes can change on temporal scales.’–Allman and Fitzhugh (2007)

Amid global declines in the sustainable harvest of marine fisheries [1], signs of rebuilding are increasing, especially in the northern hemisphere. While recovery from overfishing has proven to be a slow process confounded by ecological and socioeconomic factors [1, 2], as of 2013, twenty-eight of the 44 overfished populations in the U.S. indicate progress toward reaching sustainable population sizes [3]. One such fishery, collapsed but now rebuilding, is the northern Gulf of Mexico (Gulf) red snapper (*Lutjanus campechanus*) stock [4, 5].

Two centers (hereafter subpopulations) of red snapper abundance exist in the northern Gulf. The largest is the western subpopulation, which occurs in the northwestern Gulf off Louisiana and Texas, and is considered to be the historical center of abundance [6], while the eastern subpopulation occurs off Alabama and Florida and is much smaller [7]. Red snappers are a reef-associated species that support commercial and recreational fisheries; user conflicts make management of this species among the most controversial in U.S. waters [8].

Across the northern Gulf, from Florida to Texas, red snappers are currently managed as a single stock. While the two subpopulations in the northern Gulf are not genetically distinct [9], demographic differences exist in sizes at age and maturation rates [10–12]. It has been suggested that these demographic differences may be adaptive responses to factors including differing fishing mortality rates, habitat complexity including numbers of deployed artificial reefs versus natural reef outcroppings, and regional population sizes [10–12].

Red snappers age-10 and older are infrequently captured in the Gulf, and fish age-6 and younger comprise over 90% of the stock in the north-central and northwestern Gulf [8, 13–16]. Significant declines in stock size [4], and the removal of older individuals [13, 14] appears to have resulted in a phenotypic stress responses, including early maturation [11], faster growth [10, 12, 14] and smaller sizes-at-age [12, 14]. This is problematic because increased reliance on younger, smaller fish limits reproductive potential and resilience, and slows recovery from overfishing [17].

**Fig 1:**
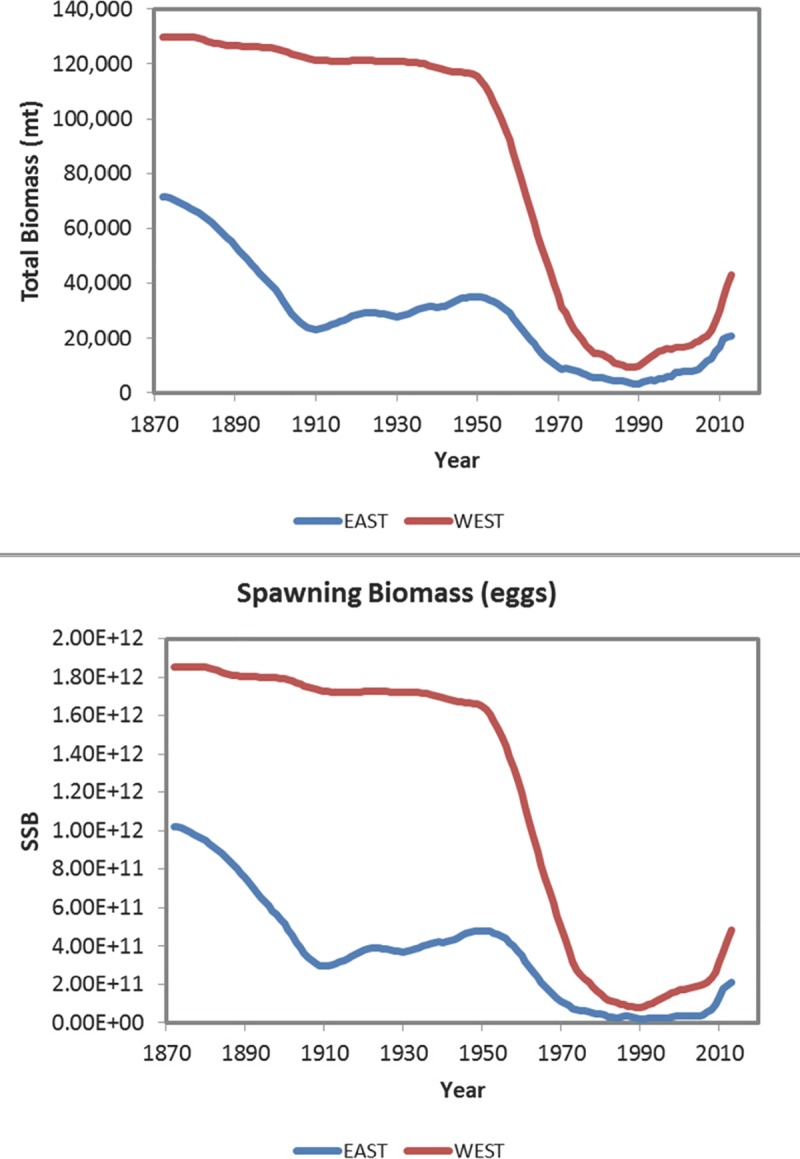
Temporal progressions of Gulf red snapper stock biomass (million tons) and spawning stock biomass (SSB) from the 1880’s to 2015. Landings are indicated east and west of the Mississippi River. Figure obtained from SEDAR 31 Assessment Update [16].

Today, the Gulf red snapper stock is rebuilding. Overfishing is no longer occurring [15, 18], and an increase in biomass is evident (Fig 1) [5, 16]. Spawning potential ratio (SPR), a benchmark used to assess stock condition and a proxy for maximum sustainable yield, has increased to ~13% and ~22% in the eastern and western Gulf, respectively [16]. A rebuilding target of 26% SPR for the U.S. Gulf, (i.e., 26% spawning potential ratio for both areas combined) has been in place for more than 20 years; this benchmark rebuilding target must be reached by 2032 in accordance with regulatory Amendment 22 to the Reef Fish Fishery Management Plan and the Sustainable Fisheries Act [5, 13]. Although overfishing is no longer occurring, the stock remains overfished with a highly truncated age structure [5, 12, 16].

In this study, we investigated whether a suite of reproductive characteristics differed between time periods before and during recovery from overfishing, a time frame of ~10 years. We assume the ‘period of recovery’ began in 2008 (when the ‘undergoing overfishing’ status of the stock was declared officially over [18]) and has an open end date, which will be determined when the US Gulf-wide rebuilding target of 26% SPR is reached. Gonadosomatic indices, sizes- and ages-at-maturity, batch fecundity, spawning frequency, annual fecundity and indices of reproductive importance were calculated and compared to determine whether each of these reproductive characteristics have remained stable over time, especially in light of recent signs of stock recovery. Additionally, because the red snapper stock differs demographically, reproductive characteristics were evaluated before and during recovery both east and west of the Mississippi River (demarking the two subpopulations previously described).

## Methods

### Sampling

This study was carried out in strict accordance with the recommendations in the Guidelines for the Use of Fishes in Research by the American Fisheries Society. The study was approved by the Institutional Animal Care and Use Committee (Protocol 08–036) in the Division of Laboratory Animal Medicine in the Louisiana State University School of Veterinary Medicine. Red snappers retained for sampling were euthanized in an ice slurry containing clove oil in amounts adjusted for the number of fish in the slurry in a given time period. Scientific Collection Permit F/SER24:SG SER08-003. The LOA recognizes the activities in accordance with the definitions and guidance at 50 CFR 600.10 and 600.745. As such, the proposed activities are not subject to federal regulations in 50 CFR 622 or Essential Fish Habitat requirements in 450 CFR 925 developed in accordance with the Magnuson-Stevens Fishery Conservation and Management Act. This LOA was signed by Roy Crabtree, the Southeast Regional Administrator of the National Marine Fisheries Service.

Red snapper were collected from neritic waters at the two known subpopulation centers in the northern Gulf: east (off Alabama) and west (off Louisiana) of the Mississippi River [7]. Red snapper in these two regions are demographically distinct [13, 16] and thus were considered separately to minimize spatial bias. Sampling occurred ~10 years apart (1999–2001 and 2009–2010) during protracted spawning seasons, which last from April to October [19–22].

**Fig 2:**
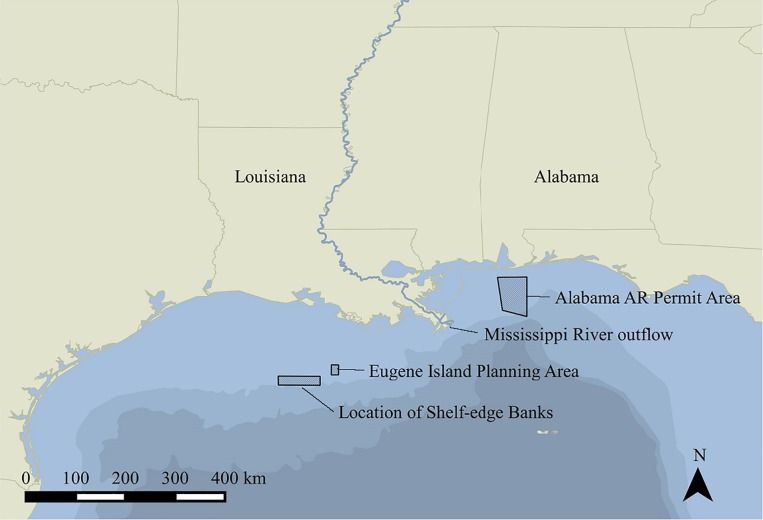
Map of study sites. Sampling occurred east (off Alabama) and west (off Louisiana) of the Mississippi River. Map made using QGIS (2016). Mississippi River layer courtesy of USGS Watershed Boundary Dataset, available at nhd.usgs.gov/wbd.html.

During the first sampling period (1999–2001), red snappers were caught by hook and line and sampled from May to October at recreational fishing docks on Dauphin Island, Alabama, and in Port Fourchon, Louisiana. Fish from Alabama waters were caught on artificial reefs (ARs) within the Alabama General Permit Areas (Fig 2), located between 18 and 85 km south-southeast of Dauphin Island, Alabama. Louisiana fish were caught at artificial structures consisting primarily of standing and toppled oil and gas platforms. In 1999 and 2000, no less than 600 fish were sampled per region, per year. In 2001, at least 300 fish per region were collected. To include data on larger specimens, ~50 fish ˃7 kg wet total weight (TW) were collected per region from fishing tournaments each year. Minimum size limit restrictions on the recreational fishery were 15” and 18” in 1999 and 16” in 2000–2001. Fish smaller than the minimum size limit were obtained under a NOAA Fisheries Letter of Agreement (LOA) by hook and line.

During the second sampling period in 2009–2010, red snappers were again collected from recreational fishing docks at Dauphin Island and Port Fourchon, as well as by vertical longline from natural shelf-edge banks (Fig 2) off the Louisiana coast, and on petroleum platforms in the Eugene Island Oil and Gas Lease Block (Fig 2); all natural bank and lease block sites were between 153 to 237 km southeast of Port Fourchon (these areas were not directly targeted in 1999–2001). At recreational fishing docks, a minimum of 200 fish was targeted per region each year. In 2009, sampling from the recreational fishery occurred as planned; additionally, 637 individuals were collected offshore. Sampling efforts in 2010 were disrupted by the Deepwater Horizon oil spill, which began on April 20 off Louisiana; the well was capped on September 19 [23]. The spill resulted in fishery closures for Alabama and Louisiana; however, 212 individuals were collected with a NOAA Fisheries LOA by vertical longline off the Louisiana coast in 2010

Similar collection methods were used during both sampling periods. Upon collection, wet TW (nearest 0.01 g), fork length (FL) (nearest mm) and total length (TL) (nearest mm) was measured and recorded. Gender was determined macroscopically whenever possible. Sagittal otoliths were removed and later processed for age analysis following Cowan et al. [24].

Because reproductive potential is limited by egg production in red snappers, only females were analyzed for oocyte cell development during both sampling periods. Ovaries were removed and visceral and adipose tissues were trimmed away. All gonads sampled from recreational catches were kept on ice in bags and transported to the laboratory where they were weighed (nearest 0.01 g) and fixed in 10% formalin for a minimum of 14 days. Gonads collected during extended (5–10 day) cruises on the Louisiana shelf were placed in plastic freezer bags and kept frozen until transported to the laboratory, where they were thawed, blotted dry, weighed (nearest 0.01 g) and fixed in 10% formalin for a minimum of 14 days.

### Tissue processing & histology slide preparation

Post-fixation, a 2-mm thick tissue subsample was dissected from one of six subsections comprising each ovary; the subsection chosen for sample extraction was determined with a single roll of a six-sided die. Subsamples were vacuum infiltrated, embedded in paraffin and sectioned (3–4 μm thickness). Embedded sections were mounted on microscope slides, stained and counterstained with hematoxylin and eosin, and coverslips were applied.

### Oocyte stage analysis and reproductive phases

Histological sections were examined microscopically at 40x to 100x magnification. The four major stages of oocyte development for heterochronal fishes are defined by Wallace and Selman [25]: primary growth (PG), cortical alveoli (CA), true vitellogenesis, and oocyte maturation [25].

In effort to incorporate more recent standardized terminology for teleost reproductive development into this study, seven additional oocyte developmental stages were also recognized: 3 subphases of vitellogenesis (primary, secondary and tertiary) and 4 subphases of oocyte maturation (germinal vesicle migration, germinal vesicle breakdown, yolk clarification and hydration) [25–27].

**Fig 3:**
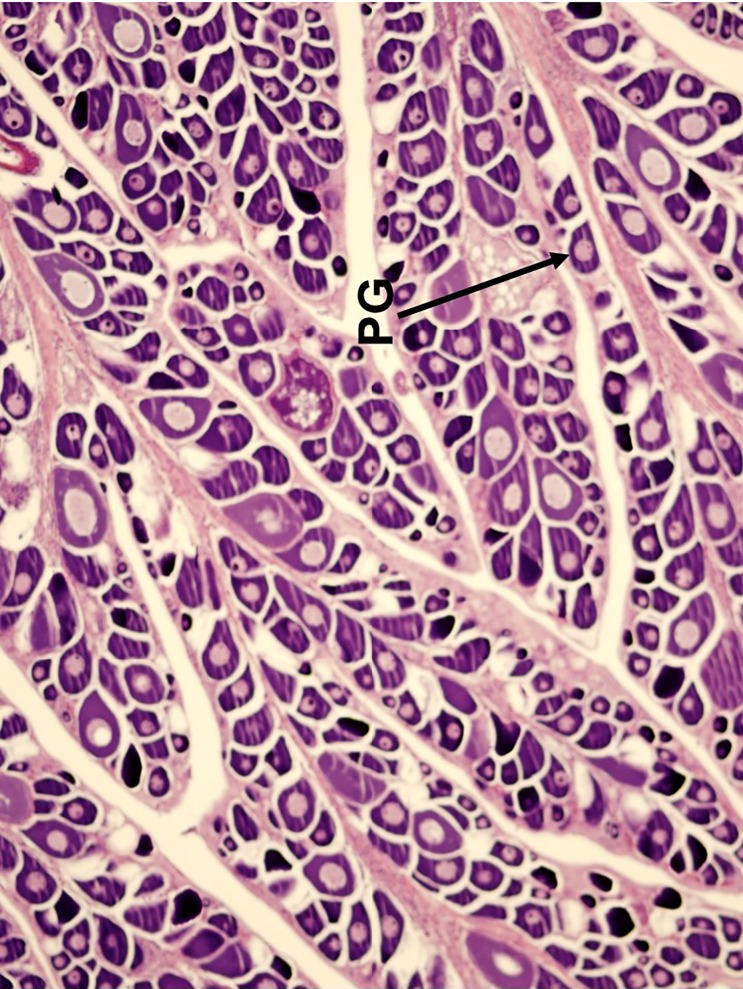
Histological image of the ‘immature’ reproductive phase in the red snapper ovary. Primary growth (PG) oocytes are distributed throughout the ovary with no other oocyte development stage present.

**Fig 4:**
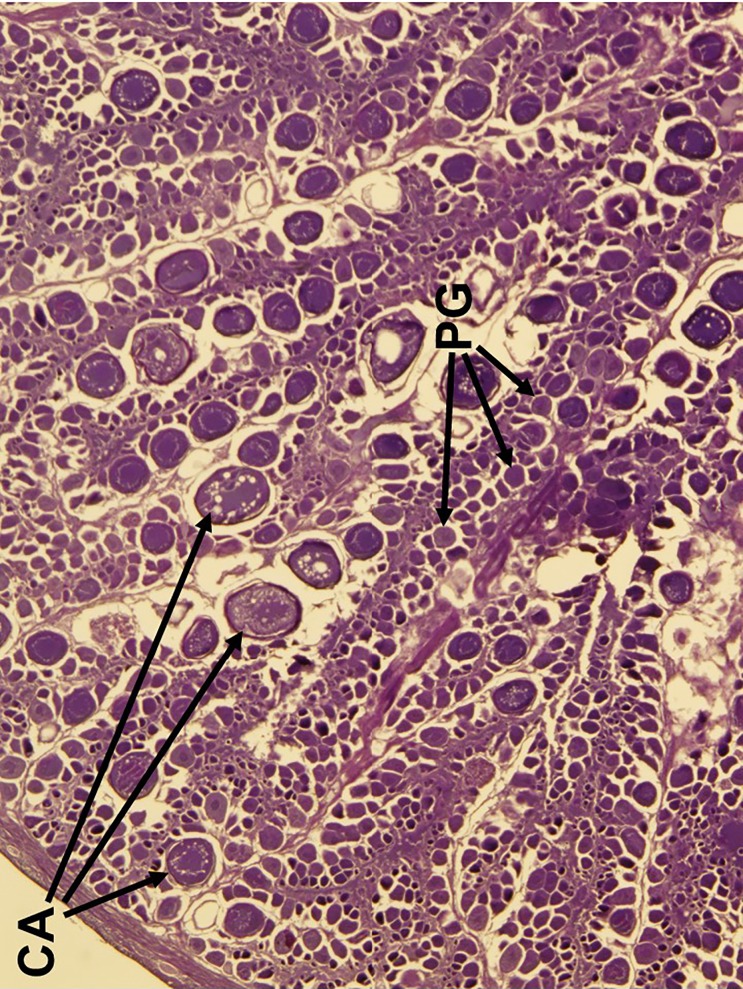
Histological image of the ‘early developing’ subphase of reproductive development in the red snapper ovary. Primary growth (PG) oocytes and cortical alveoli (CA) are the only oocytes present in the ovary.

**Fig 5:**
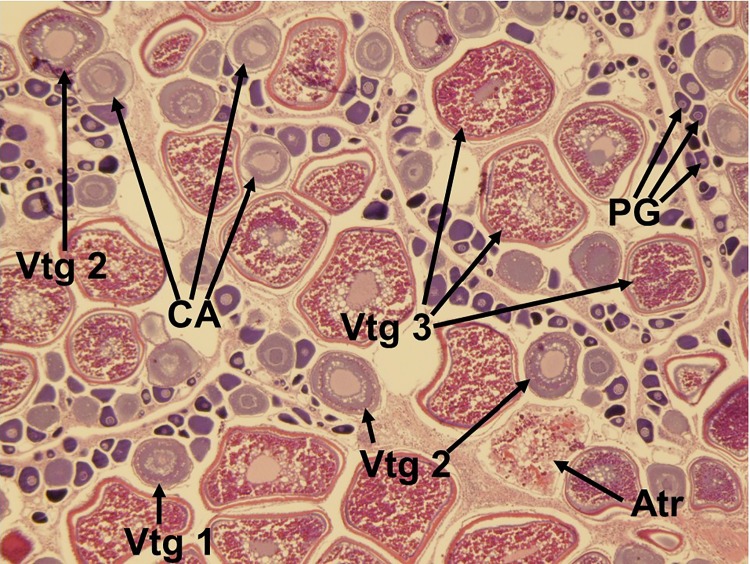
Histological image of the ‘spawning capable’ reproductive phase in the red snapper ovary. Oocyte development through the tertiary vitellogenic stage (Vtg 3) is apparent. PG = primary growth oocyte; CA = cortical alveoli; Vtg 1 = primary vitellogenic oocyte; Vtg 2 = secondary vitellogenic oocyte; Atr = atresia.

**Fig 6:**
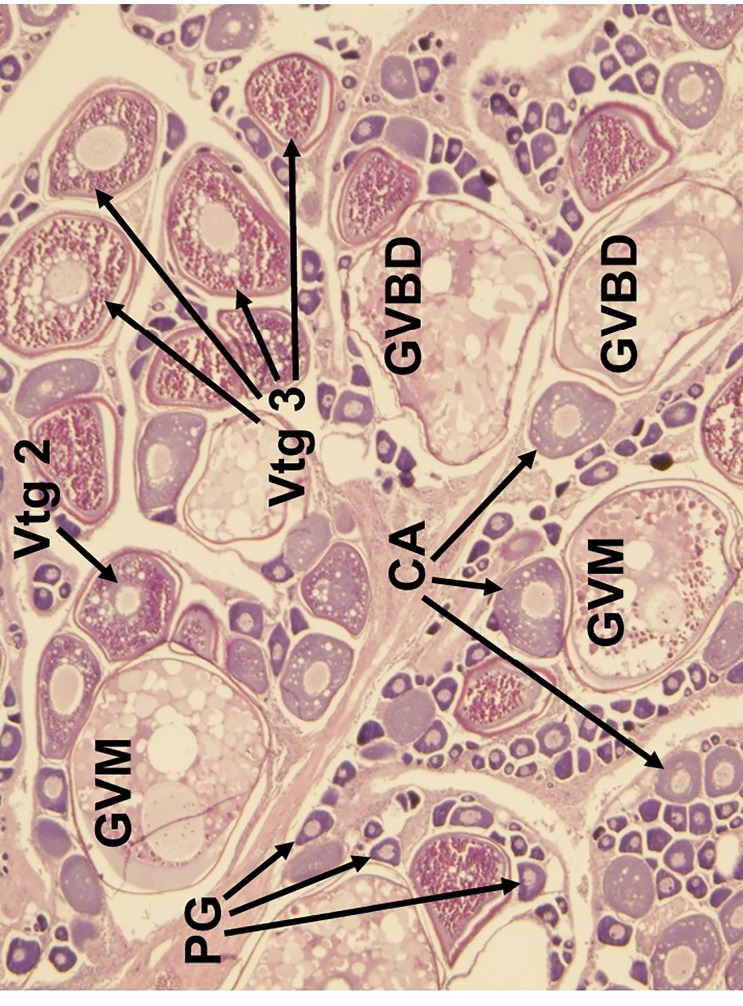
Histological image of the ‘actively spawning’ subphase of oocyte maturation in the red snapper ovary. Oocyte developmental stages occur through germinal vesicle migration (GVM) and germinal vesicle breakdown (GVBD). PG, primary growth oocyte; CA, cortical alveoli; Vtg 2, secondary vitellogenic oocyte.

**Fig 7:**
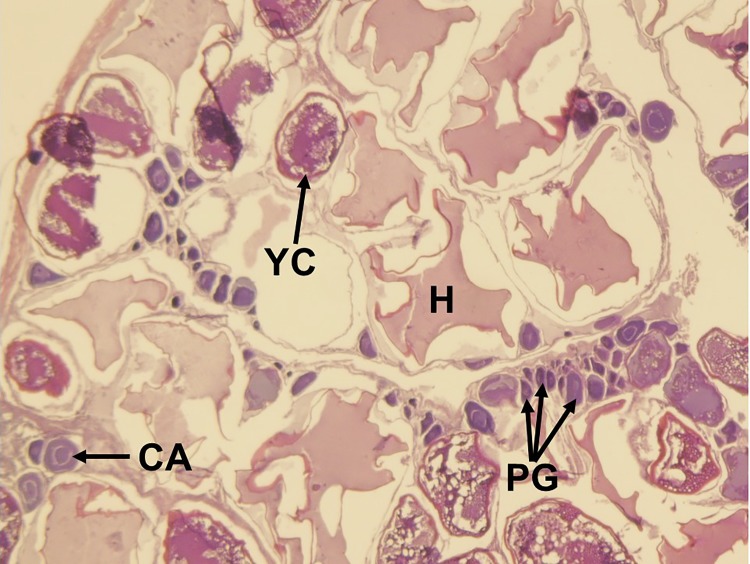
Histological image of the ‘actively spawning’ subphase of oocyte maturation in the red snapper ovary. Yolk clarification (YC) is evident. Hydrated oocytes (H) indicate imminent spawning. PG, primary growth oocyte; CA, cortical alveoli.

**Fig 8:**
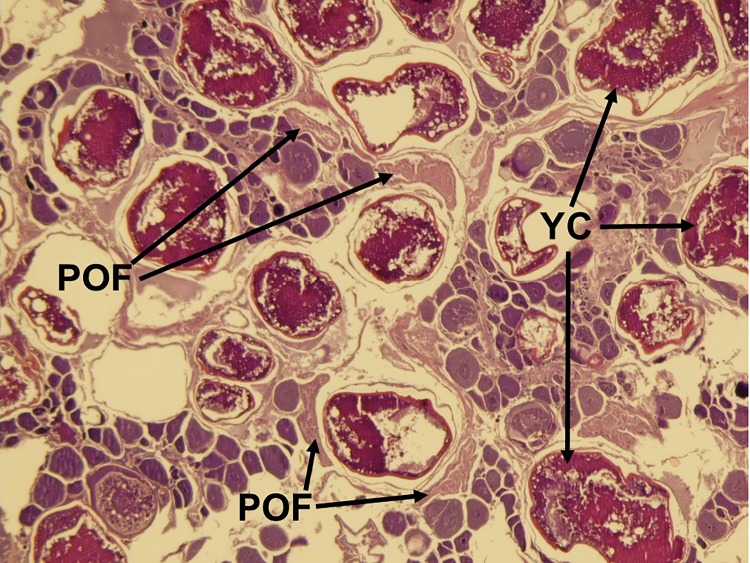
Histological image of the ‘actively spawning’ subphase of oocyte maturation in the red snapper ovary. Yolk clarification (YC) is evident. Fresh post-ovulatory follicles (POFs) indicate recent spawning.

Five reproductive phases, recently defined by Brown-Peterson et al. [26], were also considered: immature, developing, spawning capable, regressing and regenerating. The immature ovary contains only PG oocytes (Fig 3), while the developing ovary contains PG, CA, and primary and secondary vitellogenic oocytes [26] (Figs 4 and 5). The spawning capable ovary contains ‘tertiary vitellogenic’ oocytes, often referred to as ‘true vitellogenic’ or simply ‘vitellogenic’ oocytes (Fig 5) [11, 26, 28, 29]. True/tertiary vitellogenesis is the principal stage of oocyte development, as it marks the completion of yolk accretion and indicates oocytes possess receptors for the maturation-inducing hormone, thereby providing evidence that a female is physiologically able to spawn [26]. After vitellogenesis, spawning capable individuals undergo oocyte maturation, beginning with germinal vesicle migration (GVM) (Fig 6) and ending with the hydration (H) subphase just prior to ovulation [25–27] (Fig 7). One post-developmental structure, the fresh post-ovulatory follicle (POF) (Fig 8), is visible after ovulation, indicating spawning occurred ≤24 hours prior to capture [30].

It is critical to recognize that all previous reproductive studies and stock assessments of red snapper in U.S. and Mexican waters have referred to the true/tertiary vitellogenic oocyte developmental stage simply as ‘vitellogenesis,’ while the spawning capable reproductive phase has been labelled ‘maturity’ [11, 19, 21, 31–37]. As such, in this study we chose to continue use of these two terms, to remain consistent with prior research on this species.

### Gonadosomatic index

Gonadosomatic index (GSI) is the ratio of gonad weight (grams) to body weight (grams) [30]:

$$GSI=\frac{\left(Ovary\;weight\right)}{\left(Body\;weight\right)}\times 100$$

Wet gonad-free body weight was used to determine GSI values. To examine seasonal variation in spawning activity and reproductive readiness, GSI values were plotted by month.

Undamaged gonads are often difficult to obtain from recreational catches; thus, for four females sampled during 2009–2010, only one intact lobe was available. For these samples, the weight of the intact lobe was doubled to estimate undamaged ovary weight. This was feasible because two symmetric lobes comprise the red snapper ovary [33]. For GSI data collected in 1999–2001, fork length (mm) was determined from total weight using a length-weight regression for red snapper [38].

### Maturity

To distinguish immature females from those that were mature and undergoing either the developing phase of reproductive development at the start of spawning season, or the regressing phases of reproductive development at the end of spawning [26], only females sampled during the known peak spawning months for the northern Gulf (June, July and early August) [21, 33, 34] were considered for maturity estimates. Individuals were grouped by age and size class (birth year; nearest 50 mm fork length) to determine percent maturity. For fish collected in 1999–2001, insufficient data were available to determine percent maturity; therefore, percent maturity estimates at age and size from a previous study [11] were applied to obtain results for that sampling period. To compare our results for maturity-at-length with other studies, a function developed by Allman et al. [39] was used to convert TL measurements to FL when necessary.

### Spawning frequency

Spawning frequency (SF) is defined here as the intervening time between consecutive spawning events [40]. Spawning frequency was estimated using the proportion of all spawning-capable individuals for imminent (day-0) or recent (day-1) spawners using: 1) the hydrated oocyte method (H method) [30]; 2) the post-ovulatory follicle method (POF method) [30]; and 3) the time-calibrated method (TC method) [41–44]. The mean number of spawning events per reproductive season was estimated using spawning frequency estimates and a 150-day spawning season [45].

### Batch fecundity

Batch fecundity refers to the number of eggs a female produces during one spawning episode. When hydrated oocytes were present, three tissue subsamples (0.03–0.05 g each) were haphazardly excised from the ovary, weighed (nearest 0.001 g), and a glycerin spread was made. Hydrated oocytes were enumerated microscopically (10 x magnification). Batch fecundity (BF) estimates were determined gravimetrically following the hydrated oocyte method described by Hunter et al. [46]:

$$BF=\frac{\left(number\;hydrated\;oocytes\right)}{\left(tissue\;sample\;weight\right)}\times (total\;ovary\;weight)$$

The BF estimate for each fish was the mean of the estimates derived from three subsamples.

### Annual fecundity

Annual fecundity (AF) estimates were determined according to methods described by Nieland and Wilson [47]:

$$AF=\frac{\left(number\;of\;days\;in\;reproductive\;season\right)}{SF}\times BF$$

A 150-day spawning season [34] and the time-calibrated method for estimating spawning frequency was incorporated into AF estimates. The time-calibrated method was used because it is the only method that accounts for both day-0 and day-1 spawners [48].

### Index of reproductive importance

An index of reproductive importance (IRI) was developed to identify the most significant age group(s) contributing to the Gulf spawning stock [49]. Ages 0 to 8 and ≥ 9 years were evaluated using AF estimates (this study), percent maturity (this study; [11]), and NOAA estimates for annual stock age composition (% n) updated through 2013 [16]. Annual stock age composition (% n) estimates are defined as the proportion of the Gulf-wide red snapper stock that each age group comprises each year. IRI was determined as follows:

$${IRI}_{age\;i}=\frac{{\left[\left(AF)\times (\%\;n)\times (\%\;Mature\right)\right]}_{age\;i}}{\sum_{i=0}^{\geq 9}\left[\left(AF)\times (\%\;n)\times (\%\;Mature\right)\right]}$$

The IRI estimates here include the most recent %N estimates for both sampling periods in this study[16].

For each sampling period, regional reproductive estimates were combined to increase sample size among age groups. Fecundity data collected in 2009–2010 were derived from fewer females (n = 35) when compared with that from 1999–2001. Therefore, to increase sample size, we included fecundity, maturity and time-calibrated spawning frequency data for female red snapper collected from Gulf waters during the peak spawning months of 2009 and 2010 (n = 1,811) [50]; these individuals were caught off Clearwater, FL, Destin, FL, Dauphin Island, AL, Port Fourchon, LA, Galveston, TX, and South Padre Island, TX. Despite inclusion of this larger data set, fecundity data for the 2, 8 and ≥9 age groups were absent from our samples. No age-2 specimens were collected due to minimum size limits for the recreational fishery, and the two older age groups were missing due to age truncation in the stock. Thus, fecundity and SF estimates from the most recent report available [51] were used to fill these age gaps. As such, the ≥9 age group represents mean estimates for ages 9 to 40 years [51].

### Length and weight

When fork length (FL) data were not available, a linear function was used to extrapolate FL from TL [39]. Mean weights were compared using wet eviscerated body weight (EBW). Missing EBW values were predicted from wet TW using the following linear function:

EBW=0.9329×TW+15.562

This function (R^2^ = 0.9946) was based on EBW and TW data for female red snapper (n = 250) sampled from the Louisiana continental shelf [50].

### Data analysis

All statistical analyses were performed using SAS 9.3.1 (SAS Institute Inc., Cary, NC) and R 3.2.1 (R Core Team 2015) software. Analysis of variance (ANOVA) with the Tukey-Kramer post-hoc test was used to compare mean ages, lengths, weights, and GSI. For mean fork length and mean GSI, the assumption of normality was not met, however a best-estimate ANOVA was used because the sample sizes were large (fork length data, n = 2,475; GSI data, n = 1,770). Chi-square analyses were used to compare SF estimates, and when the chi square test of independence assumption of sufficient sample size was not met, Fisher’s exact test was used. Because BF data did not meet the assumption of normality, the non-parametric Mann-Whitney U-test was used to compare BF means. Linear regression was used to examine relationships between natural logarithm (log_e_) transformations of GSI and BF at size and age. Analysis of covariance (ANCOVA) was used to test for differences among regression relationships. For all tests, α = 0.05. We compared four sample groups, denoted hereafter as: EG1, Eastern Gulf Sampling 1999–2001; WG1, Western Gulf Sampling 1999–2001; EG2, Eastern Gulf Sampling 2009–2010; WG2, Western Gulf Sampling 2009–2010.

Additionally, in consideration of the possibility of fish ages nfluence on our results, as well as energetic trade-offs between growth and reproduction [52–54], reproductive estimates were assessed for 3 age categories specific to discrete somatic growth rates for GOM red snapper [12]: ≤5 years, when rapid linear growth occurs; 6–8 years, when growth rate begins to decrease; and ≥9 years, when growth rates decline substantially. Because the minimum age of spawning capability occurs at age-2 for Gulf red snapper [34, 55], and only reproductively capable individuals were considered for reproductive analyses in this study, the ≤5 years age category described above includes only 2–5 year olds and is thus referred to as the ‘2–5 years’ age category.

## Results

A total of 2,547 female red snappers were sampled: 1,947 during 1999–2001 (EG1 n = 1,033; WG1 n = 908) and 606 during 2009–2010 (EG2 n = 120; WG2 n = 486) (Table 1). Fish from the eastern subpopulation were caught almost exclusively at artificial reefs (ARs) during both sampling periods. Individuals in WG1 also were sampled almost entirely from ARs, but the majority of fish in WG2 were caught by short vertical longlines on the shelf-edge banks off the Louisiana coast (n = 413) at ARs (n = 241) and natural reef habitat (n = 172). All WG2 fish caught by the recreational fishery were from ARs (n = 73). Sex ratios for both datasets compared in this study (1999–2001 and 2009–2010) were reported in two previous publications and did not differ significantly from 1:1 during either sampling period [49, 56].

**Table 1:** Monthly catches of female red snapper (*Lutjanus campechanus*) collected from the northern Gulf of Mexico east and west of the Mississippi River outflow.

Region	Year	Apr	May	Jun	Jul	Aug	Sept	Oct	Total
East	1999	-	75	41	149	92	25	4	386
East	2000	-	74	64	115	96	34	-	383
East	2001	24	56	35	134	15	-	-	264
East	2009	-	-	120	-	-	-	-	120
East	2010	-	-	-	-	-	-	-	0
West	1999	39	82	85	85	49	-	-	340
West	2000	41	84	51	96	41	81	3	397
West	2001	40	56	56	19	-	-	-	171
West	2009	51	-	149	176	28	-	-	404
West	2010	32	-	-	50	-	-	-	82
Both	All	227	427	601	824	321	140	7	2547

Red snapper collected during EG1 ranged from 237 to 916 mm FL and from 1 to 34 years of age (Table 2). Fish in WG1 ranged from 114 to 910 mm FL and from 2 to 37 years of age. Individuals in EG2 ranged from 399 to 827 mm FL and from 3 to 16 years of age. Fish in WG2 ranged from 219 to 758 mm FL and from 2 to 11 years of age. Because age, length and weight correlates positively with egg production in female red snapper [57], the means of those variables were compared among the four groups (Table 2).

**Table 2:** Mean age (years), fork length (millimeters) and total wet weight (kilograms) of female red snapper (*Lutjanus campechanus*) sampled from the northern Gulf of Mexico (Gulf) in 1999, 2000, 2001, 2009 and 2010.

**Age**	**EG1**	**EG2**	**WG1**	**WG2**	**All**
n	964	108	886	471	2429
Min Age	1	3	2	2	1
Max Age	34	16	37	11	37
Mean Age	5.0	4.8	4.8	4.4	4.8
SE	0.1^A^	0.1^A^^B^	0.1^A^^B^	0.1^B^	0.1
**FL**	**EG1**	**EG2**	**WG1**	**WG2**	**All**
n	976	109	905	485	2475
Min FL	237	399	114	219	114
Max FL	916	826.9	910	758.1	916
Mean FL	509.9	563.9	515	478.9	508.1
SE	4.8^A^	6.6^B^	4.7^A^	4.5^C^	2.7
**TW**	**EG1**	**EG2**	**WG1**	**WG2**	**All**
n	947	92	901	449	2389
Min TW	0.986	0.239	0.445	0.193	0.193
Max TW	11.863	13.785	14.489	6.645	14.489
Mean TW	3.045	2.963	2.984	1.847	2.764
SE	0.044^A^	0.290^A^	0.094^A^	0.057^B^	0.053

FL, fork length; TW, total weight; EG1, eastern Gulf 1999–2001; EG2, eastern Gulf 2009; WG1, western Gulf 1999–2001; WG2, western Gulf 2009–2010; All, all data combined; n, sample size; Min, minimum; Max, maximum; SE, standard error.

^A,B,C^ Similar superscript letters indicate no significant difference in mean values between sample groups, according to Analysis of Variance (ANOVA) results and the Tukey honest significant difference (HSD) post-hoc test.

Age data were available for 2,429 females. Overall, females ranged from 1 to 37 years old, and the average individual sampled (± standard error) was 4.8 ± 0.1 years old. The majority (77.1%) of individuals caught were 3, 4 and 5 years old (age-3 = 25.5%; age-4 = 34.3%; age-5 = 17.3%). The only significant difference in mean age was that females in WG2 were significantly younger (mean = 4.4 ± 0.1 years) than fish in EG1 (mean = 5.0 ± 0.1 years) (p = 0.0184).

Length data were available for 2,475 females. Females ranged from 114 to 916 mm FL. To compare lengths among sample groups, fish were grouped into 50 mm size classes. With all catches combined, the majority of females (52.4%) were 375–524 mm FL (375–424 mm FL = 19.4%; 425–474 mm FL = 19.0%; 475–524 mm FL = 14.1%). Significant differences in mean fork length were detected among samples in all groups, except between EG1 and WG1 (p = 0.8470). Fish in EG2 were significantly longer (mean = 563.9 ± 6.6 mm FL) than fish in all other sample groups (p <0.0001 to 0.0020), while WG2 fish (mean = 478.9 ± 4.5 mm FL) were significantly shorter than all other groups (p <0.0001 to 0.0002).

Weight measurements were obtained for 2,389 females. Eviscerated body weight ranged from 0.193 to 14.489 kg. Fish were grouped into nearest kilogram (kg) weight classes for comparison. The 1.000 kg (37.5%) and 2.000 kg (27.4%) weight classes accounted for 65.0% of all catches combined. Fish in WG2 were significantly lighter (mean = 1.847 ± 0.057 kg) compared to all other sample groups (p <0.0001 to 0.0002). No other significant differences in mean EBW were found.

### Reproductive analyses

Examination of oocyte developmental stages was possible for 2,180 females. Of these, 81% exhibited vitellogenic oocytes (n = 1,770), indicating sexual maturity. Only sexually mature females were considered for reproductive analyses.

#### Spawning seasonality

**Fig 9:**
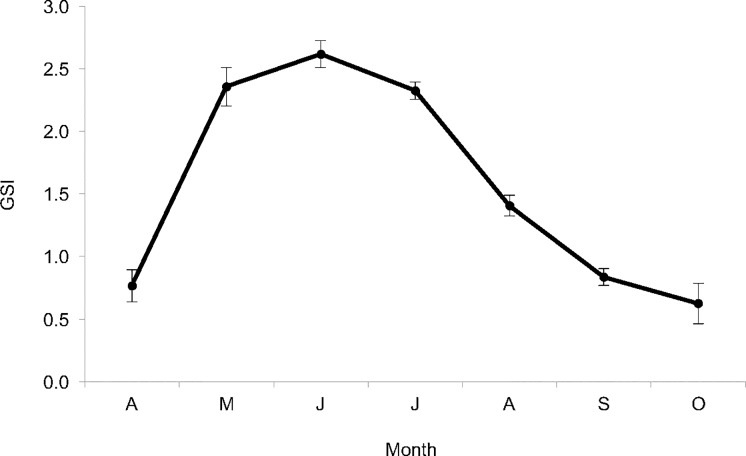
Mean monthly GSI for female red snapper (*Lutjanus campechanus*) collected from the Gulf of Mexico off Alabama and Louisiana in April through October of 1999, 2000, 2001, 2009 and 2010 (n = 1,770). Error bars represent standard error.

Throughout the spawning season, asynchronous oocyte development was confirmed microscopically through the concurrence of multiple oocyte development stages within the ovaries. With all samples combined, GS1 values ≥1 confirmed peak spawning activity [30] from May to August (Fig 9; S1 Table). Mean GSI >0.5 but <1 in April and October indicated the regenerating and regressing phases of the reproductive cycle [26]. In this study, the majority of GSI data (88.3%) were collected in May through August (May = 10.4%; June = 23.3%; July = 38.8%; August = 15.8%), when peak spawning is known to occur [21, 33, 58].

**Fig 10:**
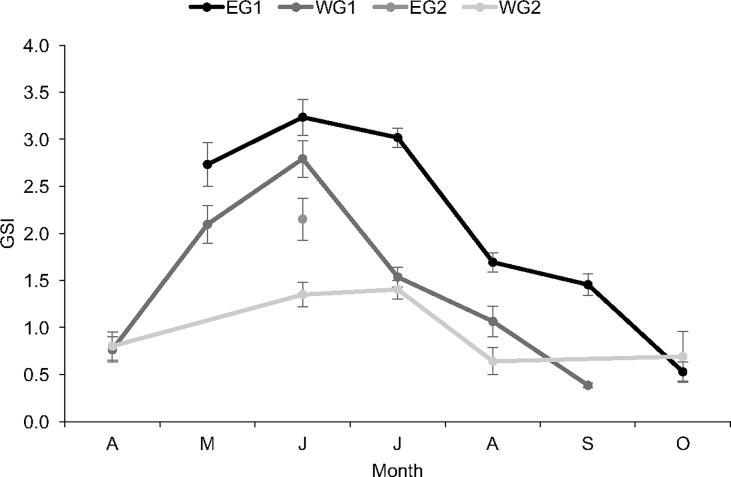
Mean monthly gonadosomatic index (GSI) values for four sample groups of female red snapper. Red snappers were sampled from the eastern Gulf of Mexico from 1999 to 2001 (EG1) and in 2009 (EG2); sampling in the western Gulf occurred from 1999 to 2001 (WG1) and from 2009 to 2010 (WG2). Error bars represent standard error.

Mean GSI was tested among the four sample groups by month (May, June, July and August). Temporal differences in spawning seasonality were observed. In both the eastern and western Gulf, mean GSI was lower among individuals sampled in 2009–2010, when compared with conspecifics sampled in 1999–2001. In the eastern Gulf, mean GSI was significantly reduced in 2009–2010 during June, when compared with fish sampled in 1999–2001 (Fig 10; S2 and S3 Tables); June was the only month when GSI data were available for fish sampled in the eastern Gulf in 2009–2010. In the western Gulf, mean GSI was also significantly lower in 2009–2010 during June, when compared with fish sampled in 1999–2001 (Fig 10; S2 and S3 Tables); a slight reduction in mean GSI was also apparent in July and August in 2009–2010, when compared with 1999–2001. Some regional differences in spawning schedules were also reflected in these GSI estimates. Mean GSI was generally greater in the east (Fig 10, S2 Table). During 1999–2001, mean GSI was larger in the east when compared with the west from May to September (Fig 10, S2 Table); these differences were significant in May, July, August and September. Similarly, during 2009–2010, mean GSI was slightly greater in the east; however, no significant difference was detected.

**Fig 11:**
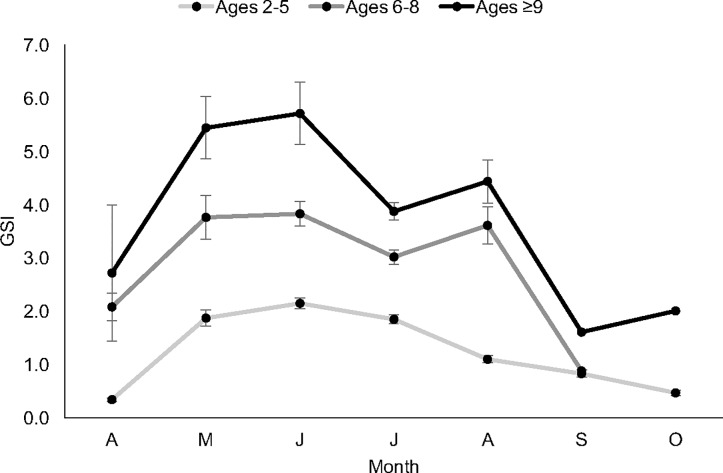
Mean monthly gonadosomatic index (GSI) values for three age groups of female red snapper. Red snappers were sampled from the Gulf of Mexico off Alabama and Louisiana in 1999, 2000, 2001, 2009 and 2010 (n = 1,495). Age groups correspond with discrete somatic growth rates at age (years) for Gulf red snapper [12]. Error bars represent standard error.

Mean GSI was also examined among the previously described growth rate-associated age groups specific to northern GOM red snapper [12]. The youngest spawning group (2–5 year olds) consistently exhibited significantly lower mean GSI values across all peak spawning months (May-August), when compared with the two older age groups (Fig 11; S4–S6 Tables). In May, June and August, 6–8 year olds displayed a slightly lower mean GSI when compared with the ≥9 age group, but no significant differences were found (p-values: 0.3843, 0.0950, and 0.8802, respectively); in July, mean GSI was significantly reduced for 6–8 year olds when compared with the ≥9 age group (p = 0.0106) (S5 and S6 Tables).

In consideration of the detected dissimilarities in mean GSI-at-age, mean GSI among the four sample groups (EG1, WG1, EG2, and WG2) was tested separately for 2–5 year olds and for ≥6 year olds. Individuals in the 6–8 and ≥9 years age groups were tested separately only for the month of July, when mean GSI was significantly different between those two age groups. From May to August combined, no temporal differences in mean GSI were found in either the eastern or western Gulf among 2–5 year olds (S7 and S8 Tables; S1 Fig). Similarly, among ≥6 year olds, temporal disparities in mean GSI were neither detected in the eastern nor western Gulf (S7 and S8 Tables; S2 Fig). Conversely, significant regional differences in mean GSI were found. During both sampling periods, mean GSI was significantly greater in the eastern Gulf when compared with the west (S7 and S8 Tables; S1 Fig).

Among individuals sampled in July only, mean GSI results for 6–8 year olds were similar to those found in the May to August combined data: no temporal differences in mean GSI were detected. Mean GSI was significantly larger in the eastern Gulf when compared with the west (S9 and S10 Tables). On the contrary, no regional difference in mean GSI was found among individuals ≥9 years old (S10 Table).

Finally, general relationships between GSI and fish size and age were examined among individuals collected from May to August. The raw GSI data best fit an exponential relationship with fork length (n = 1,495; R^2^ = 0.2486) (S3 Fig) and an asymptotic function with age (n = 1,496; R^2^ = 0.1686) (S4 Fig). To meet the assumptions of regression, GSI data were log_e_ transformed. Highly significant positive linear relationships were evident when log_e_ GSI was plotted against log_e_ fork length (S5 Fig) and log_e_ age (S6 Fig; Table 3).

**Table 3:** Best-fit regression relationships between gonadosomatic index and length (n = 1,495) and age (n = 1,496) among female red snapper (*Lutjanus campechanus*) collectively sampled from the northern Gulf of Mexico off Alabama and Louisiana in May through August of 1999, 2000, 2001, 2009 and 2010.

Linear Function	p	R^2^
Log_e_(GSI) = 1.7556 * Log_e_(L)– 10.5888	<0.0001	0.2469
Log_e_(GSI) = 0.9118 * Log_e_(A)– 0.9717	<0.0001	0.2004

Log_e_, natural logarithm; GSI, gonadosomatic index; L, fork length (mm); A, age (years); p, p-value; R^2^, coefficient of determination.

#### Maturity

**Fig 12:**
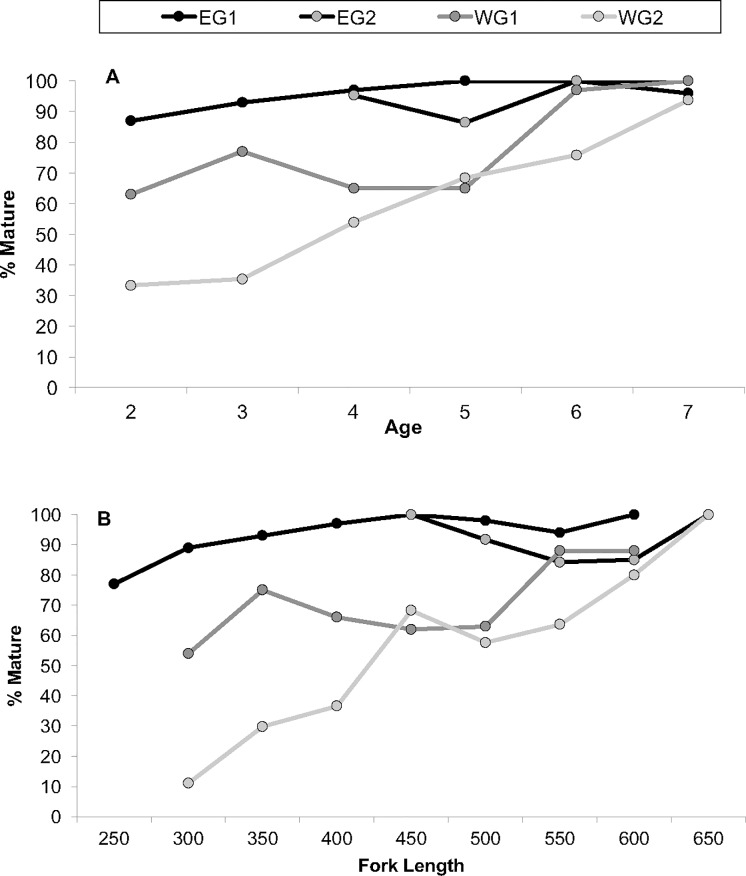
**Percent maturity at A) age (years) and B) fork length (millimeters) for female red snapper.** Red snappers were collected from the eastern Gulf of Mexico from 1999 to 2001 (EG1) and 2009 to 2010 (EG2) and from the western Gulf of Mexico from 1999 to 2001 (WG1) and 2009 to 2010 (WG2). For Fig 12A, one age-2 mature female and one age-3 mature female, both sampled from the east in 2009, was excluded due to a small sample sizes for those age groups. For Fig 12B, one mature female sampled in the east in 2009 in the 400 mm FL size group is excluded due to minimal sample size.

All sexually mature females were ≥2 years of age. Some considerable temporal differences in percent maturity-at-size and age were observed, especially in the west, where fractions of mature individuals in the 2, 3, 4 and 6 year age classes declined by 11 to 42 percentage points over time. Declines were most notable among 2- and 3-year-olds, for which the fractions of mature fish were reduced by 30 and 42% in 2009–2010 when compared with 1999–2001 (Fig 12A; Table 4). Percent maturity-at-length also declined over time in the west, especially among fish ranging from 275 to 424 mm FL, for which percent maturity was reduced by 29-to-45% during 2009–2010 as compared to 1999–2001 (Fig 12B; Table 4); a 24% reduction in the fraction of mature individuals was also observed for females of 525–574 mm FL. In the east, fractions of mature fish were similar between both sampling periods for the 4, 6 and 7-year age classes (≤ 4 percentage point difference), but 5-year-olds exhibited a reduction in maturity of 14% between 1999–2001 and 2009–2010 (Fig 12A; Table 4). Data for mature individuals in the 2- and 3-year age classes were not available for EG2. Percent maturity-at-length was similar among fish of 425–474 mm FL but decreased slightly between the two sampling periods in the east among fish of 475–624 mm FL (6 to 15 percentage point decline in fraction mature) (Fig 12B; Table 4). Data for mature fish <425 mm FL were unavailable for EG2.

**Table 4:** Percent maturity at A) age (years), and B) fork length (millimeters), for female red snapper (*Lutjanus campechanus*) sampled from the Gulf of Mexico off Alabama (eastern Gulf) and Louisiana (western Gulf) in 1999, 2000, 2001, 2009 and 2010.

	**EG1**		**EG2**		**WG1**		**WG2**	
**A. Age**	**n**	**% Mature**	**n**	**% Mature**	**n**	**% Mature**	**n**	**% Mature**
1	8	0	-	-	-	-	-	-
2	55	87^a^	1	100*	16	63^a^	6	33
3	75	93	1	100*	117	77	96	35
4	200	97	43	95	99	65	128	54^a^
5	86	100^b^	59	86	54	65	95	68
6	29	100	2	100 ^b^*	34	97	29	76
7	25	96	2	100*	8	100 ^b^	16	94
8	-	-	1	100*	-	-	5	100 ^b^
≥9	-	-	1	100*	-	-	5	100
	**EG1**		**EG2**		**WG1**		**WG2**	
**B. FL**	**n**	**% Mature**	**n**	**% Mature**	**n**	**% Mature**	**n**	**% Mature**
225–274	13	77^a^	-	-	-	-	-	-
275–324	64	89	-	-	13	54^a^	9	11
325–374	75	93	-	-	36	75	57	30
375–424	133	97	1	100*	56	66	60	37
425–474	151	100	7	100	89	62	82	68^a^
475–524	96	98	24	92	46	63	59	58
525–574	64	94	38	84	17	88	55	64
575–624	39	100 ^b^	20	85	8	88	45	80
625–674	-	-	16	100 ^b^	-	-	19	100 ^b^
675–724	-	-	1	100*	-	-	9	100
725–774	-	-	2	100*	-	-	1	100

FL, fork length; EG1, eastern Gulf 1999–2001; WG1, western Gulf 1999–2001; EG2, eastern Gulf 2009; WG2, western Gulf 2009–2010.

^a^ 50% maturity

^b^100% maturity.

*Sample size <5.

To minimize the influence of habitat type on our estimates of percent maturity, a separate maturity-at-age analysis including only fish sampled from ARs was conducted. The only sample group to include fish sampled from NR sites was WG2. Thus, all NR individuals from that group were removed from the data, and maturity-at-age was compared between WG1 and WG2. Results indicated that exclusively at ARs, 63% of WG1 fish were mature by age-2, while only 25% of WG2 fish were mature by the same age (S7 Fig). Therefore, 50% maturity was reached by age-2 among WG1 fish, while the same benchmark was reached by age-3 for WG2. Complete (100%) maturation was reached at age-7 by both sample groups.

Maturation rates also differed between regions. During both sampling periods, maturity benchmarks were reached by younger and smaller fish in the east. Fish in EG1 reached 50% maturity before age-2.5 and before 275 mm FL, and they achieved 100% maturity by 4.5 years of age and 575 mm FL (Fig 12). Fish in WG1 reached 50% maturity before age-2.5 and before 325 mm FL, and 100% maturity was reached by 6.5 years old and 625 mm FL (Fig 12; Table 4). Size- and age-at-50% maturity could not be determined in EG2 due to minimum size limits for the recreational fishery; however, that group reached 100% maturity by 5.5 years of age and 625 mm FL (Fig 12; Table 3). Females in WG2 did not reach 50% maturity until 4.5 years of age and 475 mm TL; 100% maturity was reached at an older age (7.5 years) but similar size (625 mm FL) compared to individuals from EG2. A portion of the data in WG2 consisted of individuals sampled in early August (6.9% of the data; n = 28), a time by which the greatest peaks in spawning may have passed [59]. Removing individuals collected in August did not change the sizes or ages at which fish in WG2 reached maturity benchmarks (50% or 100%).

#### Spawning frequency

Spawning frequency (SF) was estimated for 1,770 females that were determined to be sexually mature (Table 5). Among all mature individuals sampled, 39.3% displayed signs of late vitellogenic and hydrated oocytes (n = 678), while 19.4% showed signs of fresh (≥24 hours old) POFs (n = 334). Overall, females spawned an estimated once every 2.5 to 5.2 days (Table 5).

**Table 5:** Spawning frequency (SF) estimates for female red snapper (*Lutjanus campechanus*) sampled from the Gulf of Mexico off Alabama (eastern Gulf) and Louisiana (western Gulf) in 1999, 2000, 2001, 2009 and 2010.

Source	n mature	n day-0	n day-1	SF_H_	SF_POF_	SF_TC_
EG1	935	382	224	2.4	4.2	3.1
EG2*	105	34	3	3.1	35	5.7
WG1	454	226	75	2.0	6.1	3.0
WG2	231	36	32	6.4	7.2	6.8
All	1725	678	334	2.5	5.2	3.4

n, sample size; day-0, exhibiting either late vitellogenic or hydrated oocytes; day-1, exhibiting post-ovulatory follicles; SF_H_, spawning frequency estimate based on the hydrated oocyte method; SF_POF_, spawning frequency estimate based on the POF method; SF_TC_, spawning frequency estimate based on the time-calibrated method; EG1, eastern Gulf 1999–2001; EG2, eastern Gulf 2009; WG1, western Gulf 1999–2001; WG2, western Gulf 2009–2010; All, all sampling regions and years combined.

*No fish were sampled from the eastern Gulf in 2010 due to fishery closure resulting from the Deepwater Horizon oil spill.

Spawning frequency differed between sampling periods. Spawning frequency estimates and chi-square results varied greatly among the 3 methods used to estimate SF (Tables 4 and 5). However, chi-square results for SF estimated using the TC method (SF_TC_) **indicated that fish collected during 1999–2001 spawned significantly more frequently than fish collected during 2009–2010 in both the east (p = 0.0019) and the west (p<0.0001) (Table 6**). No regional difference in SF_TC_ estimates were detected during sampling period one (p = 0.7817) or sampling period two (p = 0.4973) (Table 6).

**Table 6:** Chi-Square test p-values for spawning frequency estimates compared among female red snapper (*Lutjanus campechanus*) sampled from Gulf of Mexico off Alabama (eastern Gulf) and Louisiana (western Gulf) in 1999, 2000, 2001, 2009 and 2010. Spawning frequency was estimated using the hydrated oocyte method, the post-ovulatory follicle method, and the time-calibrated method.

**Hydrated Oocyte Method**			
**Source**	**EG1**	**WG1**	**EG2**
WG1	0.0017	-	-
EG2	0.0928	0.0013	-
WG2	<0.0001	<0.0001	0.0004
**Post-Ovulatory Follicle Method**			
**Source**	**EG1**	**WG1**	**EG2**
WG1	0.0016	-	-
EG2	<0.0001	0.0003	-
WG2	0.0009	0.3634	0.0022
**Time-Calibrated Method**			
**Source**	**EG1**	**WG1**	**EG2**
WG1	0.7817	-	-
EG2	0.0019	0.0018	-
WG2	<0.0001	<0.0001	0.4973

EG1, eastern Gulf 1999–2001; EG2, eastern Gulf 2009; WG1, western Gulf 1999–2001; WG2, western Gulf 2009–2010.

Equality of SF estimates among the four sample groups was further assessed for each of the following age groups: 2–5 years, 6–8 years and ≥9 years (S12 Table). For sampling period one, ages and sizes corresponding with SF estimates for individuals sampled were unavailable, therefore statistical comparisons could not be made. During sampling period two, chi-square and Fisher’s exact test results for SF estimated using the TC method indicated no significant regional differences among 2–5 year olds (p = 0.3229) as well as 6–8 year olds (p = 0.5675) (S13 Table). Statistical comparisons of SF among sample groups could not be made for individuals ≥9 years old due to insufficient sample sizes.

#### Batch fecundity

Overall, females produced a mean of 370,890 ± 29,280 ova (i.e. egg cells) per batch (Table 7). Batch fecundity estimates were determined for all fish exhibiting fully hydrated oocytes (n = 334). To avoid underestimation of BF, all individuals displaying simultaneous signs of hydration and POFs (n = 73), a phenomenon known as back-to-back spawning [26], were eliminated from the analyses; one extremely fecund individual (BF = 7.98*10^6^) sampled from the west in 2001 was also excluded. Thus, 260 BF estimates were compared among the sample groups (east: n = 167; west: n = 93) (Table 7). A relatively small number of hydrated fish comprised the WG2 group: only 13 individuals were found in hydrated condition, all of which were collected in 2009. Additionally, as aforementioned, the EG2 group was collected exclusively in 2009 due to restrictions placed on the fishery in 2010. Therefore, for the BF data, all fish from the second sampling period were collected in 2009.

**Table 7:** Mean batch fecundity estimates for female red snapper (*Lutjanus campechanus*) sampled from the Gulf of Mexico off Alabama (eastern Gulf) and Louisiana (western Gulf) in 1999, 2000, 2001, 2009 and 2010.

Source	n	Mean ± SE	Min	Max	95% CI
EG1	142	304997 ± 32006^A^	693	1903091	(242267, 367727)
EG2	25	283051 ± 35761^A^	45817	615702	(212960, 353142)
WG1	80	552109 ± 70608^A^	1412	2236575	(413718, 690499)
WG2	13	144386 ± 73561^B^	4631	945114	(209, 288562)
All	260	370890 ± 29280	693	2236575	(313502, 428279)

n, sample size; SE, standard error; Min, minimum; Max, maximum; CI, confidence interval; EG1, eastern Gulf 1999–2001; EG2, eastern Gulf 2009; WG1, western Gulf 1999–2001; WG2, western Gulf 2009; All, all sampling regions and years combined.

^A,B^ Similar superscript letters indicate no significant difference in mean batch fecundity estimates, according to the Mann-Whitney u-test for non-parametric data (α = 0.05).

In the west, a significant difference in mean BF was detected between the two sampling periods. Results from the Mann-Whitney U-test indicated mean BF for WG2 was significantly less than that of WG1 (p = 0.0186) (Table 7). In the east, mean BF was similar between the sampling periods (p = 0.1692). Given the low mean BF estimate for WG2, BF-at-age was plotted to determine if differences among sample groups existed. Batch fecundity-at-age was consistently smaller for WG2 when compared with the other sample groups (S8 Fig). It should be noted that all fish included in BF analyses were sampled from ARs, with the exception of one age-7 female from WG2 (615 mm FL, 3446 g EBW) collected from a NR site.

A regional difference in mean BF also was found. During sampling period one, mean BF was significantly greater in the east compared with the west (p = 0.0023) (Table 7). During sampling period two, no regional difference in mean BF was detected (p = 0.3488).

Mean BF was further evaluated among the 3 previously discussed age groups relative to red snapper somatic growth (Saari et al. 2014). Results from the Mann-Whitney U-test indicated significant differences in mean BF among all 3 age groups (all p<0.0001). As expected, mean BF was lowest among 2–5 year olds, higher among 6–8 year olds and greatest among individuals age-9 and older (S9 Fig). To determine whether age influenced mean BF results among sample groups (previously discussed, Table 7), equality of means among sample groups was tested for each age group.

Among 2–5 year olds, the Mann-Whitney U-test indicated that in the east, mean BF was significantly lower during 1999–2001 when compared with that in 2009 (p = 0.0387) (S14 Table). No temporal difference in mean BF was detected in the west (p = 0.3744) (S14 Table; S10 Fig). Regionally, mean BF among 2–5 year olds was significantly greater in the east when compared with the west during both sampling periods (1999–2001: p = 0.0005; 2009–2010: p = 0.0002) (S14 Table).

Only 4 and 5 year olds comprised the EG2 dataset for batch fecundity (S8 Fig). Thus, separate analyses were run to determine whether mean BF was similar among sample groups for 4 and 5 year olds. In both the east and the west, results from the Mann-Whitney U-test indicated no significant temporal differences in mean BF among 4 and 5 year olds (S15 Table). A regional difference was detected during 2009–2010, where mean BF was significantly lower for WG2 when compared with the EG2 group (p = 0.0009) (S15 Table). The sample size for 4–5 year olds from WG2 was low (n = 9).

Among 6–8 year olds, no data were available for EG2, and only 2 individuals were available for WG2; therefore, sampling period two was excluded from the analyses. Mean BF was similar between the east and west (p = 0.4811) among 6–8 year olds (S14 Table; S11 Fig). Mean BF comparisons among sample groups could not be made for ≥9 year olds due to insufficient sample sizes (EG1: n = 3; WG1: n = 12; EG2: n = 0; WG2: n = 0).

As is the case for indeterminate spawners [57, 60, 61], batch fecundity was positively correlated with FL, EBW and age (Table 8). Batch fecundity increased exponentially with length and weight and asymptotically with age. Batch fecundity data was log_e_-transformed to better meet the assumption of normality and to linearize relationships between BF and FL, EBW and age. Best-fit regression analyses indicated highly significant positive relationships between log_e_ BF and log_e_ FL, log_e_ EBW and log_e_ age (all p<0.0001) (Table 8). Generally, log_e_ BF correlated best with log_e_ EBW (R^2^ = 0.4821; n = 259) and log_e_ FL (R^2^ = 0.4715: n = 258) but correlated least well with log_e_ age (R^2^ = 0.4026; n = 253) (Table 8).

**Table 8:** General regression relationships between log_e_ transformed batch fecundity estimates and log_e_ transformations of fork length (millimeters), eviscerated body weight (grams) and age (years) for female red snapper (*Lutjanus campechanus*) sampled from the Gulf of Mexico off Alabama and Louisiana in 1999, 2000, 2001, 2009 and 2010.

Logarithmic Function	p-value	R^2^	n
Log_e_ (BF) = 4.3182 * Log_e_ (L) - 15.1173	<0.0001	0.4715	258
Log_e_ (BF) = 1.4241 * Log_e_ (W) + 0.8244	<0.0001	0.4821	259
Log_e_ (BF) = 2.4757 * Log_e_ (A) + 8.1680	<0.0001	0.4026	253

Log_e_, natural logarithm; BF, batch fecundity; L, fork length; W, eviscerated body weight; A, age; R^2^, coefficient of determination; n, sample size.

Analysis of covariance results testing BF regression relationships indicated all three of the regression models were highly significant (all p<0.0001). When log_e_ BF was plotted against log_e_ FL, significant differences in both slopes (p = 0.0016) and y-intercepts (p = 0.0007) were detected among the sample groups (Table 9). Likewise, regression parameters differed significantly among sample groups when log_e_ BF was plotted against log_e_ EBW (slopes: p = 0.0032; y-intercepts: p = 0.0004). Results for log_e_ BF plotted against log_e_ age indicated similar slopes among groups (p = 0.1256) but significantly different y-intercepts (p = 0.0117).

**Table 9:** Analysis of covariance (ANCOVA) results testing for equality of batch fecundity regression slopes and y-intercepts among four sample groups of female red snapper (*Lutjanus campechanus*) collected from the Gulf of Mexico off Alabama (eastern Gulf) and Louisiana (western Gulf) in 1999, 2000, 2001 and 2009: EG1, eastern Gulf 1999–2001; EG2, eastern Gulf 2009; WG1, western Gulf 1999–2001; and WG2, western Gulf 2009. Regressions tested natural log-transformed batch fecundity against natural-log transformed fork length (millimeters), eviscerated body weight (grams) and age (years).

**ANCOVA Results for Equal Slopes**						
**Model**	**n**	**df**	**SS**	**F**	**p>F**	**R**^**2**^
Log_e_ (BF)*Log_e_ (L)	258	3	17.466	5.2272	0.0016	0.5895
Log_e_ (BF)*Log_e_ (W)	259	3	15.36	4.7134	0.0032	0.6020
Log_e_ (BF)*Log_e_ (A)	253	3	7.7945	1.9282	0.1256	0.5014
**ANCOVA Results for Equal y-intercepts**						
**Model**	**n**	**df**	**SS**	**F**	**p>F**	**R**^**2**^
Log_e_ (BF)*Log_e_ (L)	258	3	19.512	5.8395	0.0007	0.5895
Log_e_ (BF)*Log_e_ (W)	259	3	20.463	6.2793	0.0004	0.6020
Log_e_ (BF)*Log_e_ (A)	253	3	15.123	3.7412	0.0117	0.5014

Log_e_, natural logarithm; BF, batch fecundity; L, fork length; W, eviscerated body weight; A, age; n, sample size; df, degrees of freedom; SS, type III sum of squares; F, F-value; p, p-value; R^2^, coefficient of determination.

#### Annual fecundity

Annual fecundity estimates were determined for all day-0 fish for which BF was estimated (n = 260). In all, red snapper females spawned once every 3.4 days, based upon SF_TC_ estimates, and produced 16.4 million ova per 150-day spawning season (Table 10).

**Table 10:** Annual fecundity estimates for female red snapper (*Lutjanus campechanus*) sampled from the Gulf of Mexico off Alabama (eastern Gulf) and Louisiana (western Gulf) in 1999, 2000, 2001, 2009 and 2010.

Source	n	SF_TC_	Mean BF	Mean AF
EG1	142	3.1	304997	14757919
EG2	25	5.7	283051	7448711
WG1	80	3.0	552109	27605450
WG2	13	6.8	144386	3184985
All	260	3.4	370890	16362794

n, sample size; SF_TC_, spawning frequency estimate based on the time-calibrated method; BF, batch fecundity estimate; AF, annual fecundity estimate; EG1, eastern Gulf 1999–2001; EG2, eastern Gulf 2009; WG1, western Gulf 1999–2001; WG2, western Gulf 2009–2010; All, all sampling regions and years combined.

#### Index of reproductive importance

Among fish from 1999–2001, IRI values indicated that age-9 and older individuals were the most significant contributors to the spawning stock, as this group alone contributed more than all other age groups combined (IRI = 0.51) (Table 11). In contrast, age-2 and -3 individuals were the least significant contributors among the spawning stock (IRI = 0.03 and 0.04, respectively). Fish aged 4-to-8 years produced IRI values that were at least twice as large as those for 2- and 3-year-olds (range: 0.08 to 0.09).

**Table 11:** Index of reproductive importance (IRI) values for Gulf of Mexico female red snapper (*Lutjanus campechanus*) sampled off Alabama and Louisiana during the spawning seasons of 1999–2001 (sampling period one) and 2009–2010 (sampling period two).

**Sampling Period One**							
**Age**	**n**	**Spawning events per season**	**BF**	**AF**	**% n**	**% Mature**	**IRI**
0	178644000	0	0	0	0.7337	0	0
1	48040967	0	0	0	0.1973	0	0
2	8772797	29.19	25253	737115	0.036	0.8810	0.0341
3	3058403	34.31	84278	2891409	0.0126	0.7540	0.0399
4	1918950	44.98	189513	8523874	0.0079	0.8484	0.0830
5	945292	36.93	464595	17157625	0.0039	0.8512	0.0825
6	531001	54.49	541252	29494527	0.0022	1.0000	0.0936
7	317031	44.59	923530	41178739	0.0013	1.0000	0.0780
8	234510	38.41	1407088	54041787	0.001	1.0000	0.0756
≥9	1006098	43.23	1974806	85375667	0.0041	1.0000	0.5134
**Sampling Period Two**							
**Age**	**n**	**Spawning events per season**	**BF**	**AF**	**% n**	**% Mature**	**IRI**
0	174361500	-	-	0	0.6692	0	0
1	58244700	-	-	0	0.2235	0	0
2	11437990	*29.00	*12000	*350000	0.0439	0.3333	0.0085
3	6614075	15	46775	467753	0.0254	0.5792	0.0114
4	4388840	23.3	95446	614512	0.0168	0.6336	0.0109
5	2395400	25	166046	996278	0.0092	0.7484	0.0114
6	1159042	25	267779	1606673	0.0044	0.7065	0.0084
7	470217	24	368483	2303016	0.0018	0.8667	0.0060
8	222392	15	*927000	*64270000	0.0009	0.8333	0.0761
≥9	1261303	9.38	*2130344	*117430313	0.0048	0.9167	0.8672

n, mean population size estimates for each sampling period based on annual estimates from the SEDAR31 Red Snapper 2015 Assessment Update [62]; % n, proportion of the stock comprised by a given age group; BF, batch fecundity estimate; AF, annual fecundity estimate, where spawning frequency was estimated using the time-calibrated method; IRI, index of reproductive importance.

* Where gaps in our data existed, recent estimates were borrowed from Porch et al. [63].

The 2009–2010 data indicated a shift in age-specific spawning contributions, when compared with estimates from 1999–2001. During 2009–2010, a large reduction in dependence on young females (ages 2–7), and an elevated dependency on older individuals (age 8 and ≥9) was observed (Table 11). Two-and-3-year-olds were 3.5 to 4.0 times less productive, 4 and 5 year olds were 7.2 to 7.6 times less productive, and 6-and-7-year-olds were 11.1 to 13.0 times less productive during 2009–2010 when compared to individuals from 1999–2001. Most strikingly, an IRI of 0.87 was observed for the ≥9 age group, indicating that age group may have provided 85–90% of total egg production (TEP) for the stock during 2009–2010. Between the two sampling periods, a slight increase in IRI (1%) over time was also observed among age-8 individuals. In contrast, declines in IRI values for the 2-to-7-year age groups were detected. For instance, data indicate that age-2 females contributed 3.4% of the stock’s TEP during 1999–2001, while fish from that same age group contributed just 0.9% of the stock’s egg production during 2009–2010; this signifies a 75% reduction in the spawning contributions of 2-year-olds between the time periods. Similarly, our data indicated a 71% drop in the spawning contributions of 3-year-olds between the time periods. Even more dramatic declines in reproductive contributions of 4-to-7-year-olds (86–92%) were found over the ~10-year span.

Interestingly, coincident with large recent reductions in IRI values among the 2-to-7-year-old age groups over time, Gulf-wide stock composition estimates indicate the number of individuals comprising each of those age groups rose by 30–129% between the two periods [16]. A 5% decline in the number of age-8 individuals occurred over this time period, while the number of fish in the ≥9 age bracket increased by 25%.

## Discussion

Reproductive biology characteristics were compared between the subpopulations and time periods, which is uncommon in studies of fish reproductive biology. In addition to time, these data also reflect differences in stock status, as 1999–2001 data were collected when red snapper were both overfished and overfishing was occurring [4, 13]. In contrast, data collected in 2009–2010 were collected during a time when the stock was rebuilding and was no longer subject to overfishing [5]. The difference in SPR between sampling periods is significant. During 1999–2001, estimated SPR was approximately 2% and 5% in the east and west subpopulations, respectively; during 2009–2010, SPR was estimated to be 3% and 10% in the respective subpopulations. On these trajectories, SPR in the west subpopulation will be close to 36% in 2032, whereas in the east subpopulation it will be ≤10%, depending on how much of the catch is reallocated to the recreational sector before 2032, given that most of the recreational fishing effort occurs in the eastern Gulf [16].

### Spawning seasonality

Oocytes are homogenously distributed throughout red snapper ovaries [33, 64]. Thus, sampling one location of the ovary is sufficient to describe all stages of oocyte development. The asynchronous progression of oocyte development was observed in both subpopulations, consistent with fishes that spawn multiple times in a single reproductive season [65]. Elevated GSI values from May to August coincided with previous reports that the spawning season extends from May/June to July/August in the northern Gulf [21, 33, 34, 59, 64].

No prior studies to date have reported GSI at size and age for red snapper. Our results indicate GSI is best explained by an exponential function of fork length, followed by an asymptotic relationship with age. These results are comparable to those from a previous report that, for red snapper, reproductive potential, measured as batch fecundity (BF), was best explained by a power function with fish length, and to a lesser extent by an asymptotic relationship with age [57]. Correlation coefficients from our study also indicated both fish length and age better fit BF estimates than GSI values; this is likely consequential of greater variability in GSI due to large fluctuations in gonad mass as oocyte development progresses toward ovulation. For instance, in the absence of significant change in relative fecundity, GSI was shown to expand up to 3.5 times < 24 hours prior to ovulation for the indeterminate spawning Japanese anchovy, *Engraulis japonicus* [66].

While GSI values were within the range of prior estimates for red snapper in the northern Gulf and U.S. coastal Atlantic [19, 33, 34, 37], fish caught during 2009–2010, the “recovery period,” exhibited lower GSI values across all spawning months, when compared with individuals from 1999–2001, the “pre-recovery period.” This trend was evident in the west, although there was some indication that a similar pattern also existed in the east. Decreased GSI during the recovery period may reflect smaller sample sizes, dissimilar age/size compositions or varying sampling locations. Generally, red snapper from the recovery period were much smaller (shorter and lighter) compared to individuals from the pre-recovery period. Because reproductive capacity is a function of maternal size [57, 67], it would be expected that GSI would be lower among the smaller females collected during the recovery period. Comparable GSIs among similar age groups during the pre-recovery and recovery periods lead us to believe that a reduced representation of larger individuals in the recovery period data likely explains the generally lower mean GSI in recent years and may be related to the rare incidence of old, large individuals in the population [8, 10, 12, 14, 21, 48].

In addition, habitat type, a factor that has been overlooked in previous studies including this one, may have played a role in our interpretations. Glenn et al. found that red snapper females collected on natural reefs and banks in the western Gulf were older (≥4 years) and had much higher GSIs during all spawning months, including periods of peak spawning, than females found on adjacent artificial reefs (≥2 years) comprised of standing and toppled oil and gas platforms [68]. It is clear that life history strategies resulting in year class dominance, coupled with changes in reproductive potential associated with habitat type, can and likely have, complicated estimates of stock productivity.

Spatial comparisons indicate that GSI estimates were greatest in the east subpopulation across all peak spawning months, indicating greater energetic investment in reproduction per body size in that region, when compared with the west. Likewise, Collins et al. also found that eastern red snapper (off Alabama/Florida) produced consistently larger GSIs when compared with western red snapper (from Louisiana/Texas) [19]. Greater energetic investment in reproduction among eastern red snapper could be indicative of an important life-history trade-off between growth and reproduction [52].

### Maturity

Consistent with numerous earlier reports, our combined data showed that sexual maturity for red snapper occurs at a minimum of 2 years of age [5, 34, 35, 37] and 250–326 mm FL [21, 31–33, 36, 37]. Previous studies indicate that 50% maturation is reached by age-2 [34, 35, 37] and 290–375 mm FL [21, 32, 36, 37, 64]. In contrast, we found that the age at which 50% maturity was reached during the recovery period was older in the west (age-3 at ARs exclusively; age-4 at AR and NR sites combined), which is similar to results reported by Glenn et al. [68]. Unfortunately, there was insufficient data to determine size and age estimates at 50% maturity in the east during the recovery period. However, all of our estimates for 100% maturity fell within ranges previously reported for red snapper, which suggest that a benchmark is reached at 5–8 years of age [34, 37] and 407–770 mm FL [21, 32–34, 37, 48].

In the pre-recovery period maturity study by Jackson et al., it was suggested that reduced sizes and ages at maturity east of the Mississippi River (off Alabama) were likely attributable to a compensatory response to overfishing referred to as juvenescence, a condition that results in faster growth rates and early maturation owing to an increase in per capita food resources among a smaller population [11]. Disproportionately high fishing mortality rates off Alabama were noted at that time [7], but fishing pressure off Alabama has declined substantially since then, although SPR remains low [5]. Other contributing factors may have included increasingly strict federal fishing regulations, different size/age structures, environmental factors, predation rates or nutrient availability between Alabama (east) and Louisiana (west) [11]. More recently, the authors of Glenn et al., one of which was involved in both studies, now puts less weight on the juvenescence hypothesis and believes that habitat type is more likely to be the most significant factor affecting reproductive potential of red snapper in the northwestern Gulf [68]. In agreement with this, our data indicated disparities in maturation rates were somewhat diminished when habitat type was similar among groups sampled.

Underlying mechanisms for differential maturation rates among subpopulations of Gulf red snapper remain unknown. To date, no studies offer conclusive evidence regarding whether spatial dissimilarities originate from ecological or evolutionary factors [11]. For a single genotype, such as red snapper, one promising tool with potential to elucidate whether changes in maturity rates are phenotypic or genetic is the probabilistic maturation reaction norm (PMRN) [69, 70]. Investigation of critical factors along a PMRN, such as the breadth and midpoint, would allow for comparisons among cohorts as well as among temporal or spatial groupings within the population, with little outside influence of environmental variability [71]. Such a tool could potentially provide a wealth of information for management of the fishery useful for determining potential yields for the stock, including short-term predictability of maturation rates based on environmentally-induced changes in growth, aid in addressing evolutionary/genotypic questions about maturation, and the potential identification of harvest-induced evolution [69, 71].

### Spawning frequency

Spawning frequency is difficult to estimate and is likely sporadic during the spawning season [48]. To account for variability in SF estimates caused by the time of day when fish were caught [45] or other factors including accelerated degradation of POFs at warm water temperatures [72], we chose to rely on SF estimates determined using the TC method in this study; this is likely the most accurate method for determining SF because it is inclusive to both day-0 and day-1 fish [34, 48], each of which are known histological spawning indicators [20]. A temporal trend toward less frequent spawning in recent years was observed. These declines in SF are attributed to reduced proportions of day-0 and day-1 females during the recovery period. Smaller sample sizes [40] and reduced size and age ranges during the recovery period may account for some of these differences, as SF is known to increase with maternal size and age in iteroparous fishes [11, 34, 57, 73–75], including red snapper [20]. On the other hand, while no studies to date have directly linked fishing pressure to changes in spawning frequency, stocks with a severely truncated age distribution are dependent upon small, younger spawners [17], which have been shown to spawn less often and over a shorter seasonal duration compared to older, larger fish [5, 20, 74, 76]. A potential regional difference in SF detected during the recovery period indicated eastern red snapper spawned slightly more often than conspecifics in the west. While this result contradicts reports that spawning frequency for red snapper is greater in the western Gulf due to the existence of larger fish in that region [20], and our findings corroborate those of Porch et al. [20], who found no regional difference in spawning frequency east or west of the Mississippi was found once age or size was considered.

### Batch fecundity

Our data support previous reports that mean BF is <1 million ova for red snapper under 8 years of age across the Gulf [19, 50]. Red snapper collected in the western Gulf displayed a relatively low mean BF estimate during the recovery period. This is probably attributed to a small sample size of hydrated females but could also result from the younger and smaller fish comprising that sample group in response to the strong 2004–2006 year classes, as it is well established that fecundity is limited by body size for red snapper [19, 33, 48, 57].

Our finding that BF increases with maternal age has been espoused in other studies [30, 60, 61, 67, 74, 75, 77–79]. We also found that BF increases exponentially with length and weight, and asymptotically with age, and size appears to have a greater effect on BF than age; these observations ally with previous research [11, 34, 57]. Inconsistent with other reports, our batch fecundity data correlated best with maternal body weight, closely followed by length. Interestingly, while the use of total weight is standard practice in fish fecundity studies [57, 80], inherent biases are associated with total weight through varying stomach and gonad masses. In our study, we attempted to eliminate these biases by using eviscerated body weight instead of total weight. The relatively large sample size of this study lends further support for our results. It should be noted, however, that a large proportion of our eviscerated body weight data was obtained by prediction from total weight values.

When age was considered, we found no temporal differences in mean BF east or west of the Mississippi River. A demographic difference in mean BF was found among young spawners (2 to 5 years of age) during the recovery period, where mean BF was greater in the east, compared with the west. Similarly, previous results generated for data collected during the pre-recovery sampling period, using ANOVA and Duncan’s Multiple Range Test, indicated individuals from the east up to 725 mm FL produced larger batches, when compared with counterparts from the west [49]. Collectively, these findings indicate that there appears to be a greater investment in reproductive output in the early years of spawning in the east, compared with the west. In our study, the small sample size in the west during the recovery period may, at least in part, account for the perceived differences in mean BF among sample groups.

### Annual fecundity

Among all females in this study, mean total annual egg production per individual was 16.4 million ova per year. This fell in range with the few other AF estimates for Gulf red snapper. Szedlmayer and Furman reported an AF of 2.9 million for 2–4 year old red snapper caught at artificial reefs off Alabama [81]. Collins et al. reported an AF range of 11.6–59.7 million ova for red snapper 3–12 years of age collected of the northwest Florida coast [33]. Those estimates are lower than that found by Woods, who reported a maximum AF of 76.6 million ova for red snapper catches off Alabama and Louisiana [49].

### Index of reproductive importance

The ages and sizes of red snapper bearing the highest reproductive importance is presumably dynamic. Results from this study indicate that females older than age-8 contributed more to the spawning stock, in terms of egg production, than all other age groups combined, while 2-year-olds contributed relatively little. With the exception of exceedingly low reproductive importance for two older age groups (6-and-7-year-olds) sampled during the recovery period, our findings generally agree with other reports that reproductive importance increases with age [49, 55]. Relative reproductive importance reportedly peaks at age-14, in the absence of fishing mortality, then steadily drops as a result of gradual declines in survivorship of older age groups [55].

Our data indicate a temporal shift toward greater dependence on older fish among thespawning stock in recent years. Most notably, red snapper older than age-8 are assigned high reproductive importance during the recovery period (IRI = 0.87); this estimate is higher than previous research suggests. Only two other studies to date have described IRI values for red snapper. For fish >8 years old, Woods reported an IRI value of 0.41 in the north central Gulf, while Kulaw reported a gulf-wide IRI estimate of 0.80 for the same age group [49, 50]. It should be noted that all IRI estimates in our study included the most recent stock composition estimates available, while Woods used older stock composition estimates available at the time [49].

Moreover, young spawners (ages 2-to-7) from the recovery period contributed far less to egg production compared to fish of similar ages sampled one decade earlier; these differences reflect decreases over that time in fecundity, rates of maturity and spawning events per season (this study). Interestingly, coincident with large temporal reductions in relative reproductive importance among the 2-to-7 age groups, stock composition estimates indicate that the numbers of individuals comprising each of those age groups actually increased by 23–71% over the timespan of our study [16]; likewise, spawning stock biomass estimates of the number of ova produced by the spawning stock essentially doubled over the same period of time (from a mean of 2.03E+11 ova during the pre-recovery period to a mean of 4.0E+11 ova during the recovery period) [16]. While it is probable that the temporal changes in IRI values reported here may be ascribed to compensatory responses associated with increased stock abundance, we cannot rule out the effects of habitat type on these findings because red snapper from natural reefs and banks were only collected during 2009–2010 and only in the western Gulf.

### Potential reasoning for differential reproductive effort and future directions

Potential sources of error in our data are primarily restricted to 2009–2010. These include reduced sample sizes, minimum size limits, severe age-truncation of fish, and differences in habitat quality among natural reefs and banks and artificial habitats in the northwestern Gulf. First, during 2009–2010, a 16-inch TL minimum size limit was imposed on the recreational fishery; this resulted in dockside data that contained few small, young fish (primarily ages up to 3 years), making it difficult to determine sizes and ages at which 50% maturity was reached. Second, age-truncation in the recreational and fishery-independent data was also apparent. To some degree, this may be related to fishing bias, as the vast majority of fish were caught at artificial reefs and oil platforms, and fish >10 years old are known to emigrate away from structure [7]. However, a lack of old, large fish is evident gulf-wide and is largely attributed to overfishing [8, 10, 13, 16]. Third, the majority of catches in the northwestern Gulf were made on natural shelf-edge banks, an area where a slower progression to maturity has been reported compared with areas where recreational fishing generally occurs [50]; in contrast, Glenn et al. (in review) reported faster rates of maturity at some shelf-edge reef and bank sites compared to those collected from artificial reefs. Furthermore, it should also be recognized that during 1999–2001, tournament fish were sampled to better understand reproductive potential among old, large females. Females of this size (˃7 kg total wet weight) were not collected during 2009–2010.

Life history traits including growth (length-at-age) and maturity provide critical information on stock composition and dynamics. These traits are phenotypically plastic in nature and fluctuate slowly over time [17]. However, vital rates including natural mortality and fishing mortality are known to influence life history characteristics through compensatory (density-dependent) processes [17]. Differences in growth rates and/or maturity schedules may indicate varying mortality rates related to local environment (food availability, habitat quality, predator abundance) or harvest practices (fishing mortality rates and/or fishing regimes) [9–12, 59, 82] and thus are useful in detecting changes in fish abundance.

Stricter federal limits on the red snapper fishery during the intervening years of this study may also bear influence on our results. The recent Gulf-wide decline in fishing mortality rates and increase in stock biomass supports the assertion that red snapper population biomass is on the rise, especially in the western Gulf [5]. As such, our observations of reduced reproductive yields during the recovery period could signal a compensatory response to increasing stock size. Despite evidence of population growth for the stock, there are only weak indications that age truncation is lessening [5, 12]. In over-exploited stocks, a truncated age structure slows recovery and is particularly challenging for long-lived species, because stock biomass must be sustained by young, small spawners [17], which haven’t yet reached an ages or sizes of maximum reproductive potential. Age truncation can also substantially reduce reproductive potential per recruit, resulting in a lower SPR (a measure of reproductive fitness) for the stock. Cooper et al. provided a clear example of the contrast between spawning stock biomass and total egg production with increasing age truncation in spotted seatrout [83]. Their results plainly show that as fishing mortality (F) increases, the number of fish surviving to older age decreases and even modest increases in F can cause extreme reductions in total egg production. It is anticipated that as the Gulf red snapper stock rebuilds, it will become comprised of a larger proportion of older individuals provided that there is sufficient escapement from the fishery [12, 84].

As with numerous other reports of demographically distinct reproductive biology among Gulf red snapper [11, 31, 32, 34, 49, 50], this study found evidence of regional distinctions in reproductive potential east and west of the Mississippi River. During 1999–2001, larger proportions of young, small individuals reaching maturity in the east were addressed previously by Jackson et al., who argued that earlier maturity in the east (off Alabama) likely resulted from a stress-induced compensatory response to severe overfishing [11]. However, a companion age-and-growth study indicated no difference in growth rates between Alabama and Louisiana red snapper at that time [10], prompting Jackson et al. to speculate that genetic selection for fast-growers led to fish reaching their physiological maximum potential growth rate in both regions [11]. As previously discussed, however, the importance of such a mechanism has been called into question by the effects of habitat type identified by Glenn et al. [68]. Finally, the number of standing oil and gas platforms peaked in the U.S. Gulf in 2001 at 4,052 and thus were increasing in number during the entirety of the first sampling period, especially in the western Gulf. In contrast, the opposite is true during 2009–2010, as oil fields retired and platforms were decommissioned and removed. By 2010 (the end of sampling period two) there were fewer than 2,500 standing platforms remaining in northwestern Gulf. In a recent review, Cowan and Rose (2016) found that the mean number of red snapper found during surveys on standing platforms off Louisiana prior to 2008 was 1,844, but ranged from 905 to 4,062 [85]. The fate of fish that had been residing on platforms post decommissioning is uncertain, but it is likely that density of red snapper increased on adjacent natural and artificial reefs as the platforms were removed. The impact of such a displacement also is uncertain, and we cannot rule out the possibility that this too affected the results of our study during 2009–2010. As such, we believe that the combination of natural and manmade factors that have the potential to affect the population dynamics of red snapper make it one of the most challenging fisheries in U.S. waters to manage.

With over half of U.S. overexploited marine fisheries currently in recovery [3], abundant opportunities for improving our understanding of the mechanisms of rebuilding are at hand. Through the recovery process, close monitoring of changes in stock production and indicators thereof, including life history, demography and reproductive potential, serve to provide fishery managers with crucial new information for better understanding the resilience of a population as well as refine forecasts and oversight of its recovery [1, 78].

## Supporting information

S1 Table Monthly gonadosomatic index (GSI) values with standard error (SE) for female red snapper (*Lutjanus campechanus*) collected from the Gulf of Mexico off Alabama and Louisiana in 1999, 2000, 2001, 2009 and 2010 (n = 1,770). (DOCX)

S2 Table Analysis of variance (ANOVA) results testing equality of mean index (GSI) values among four sample groups of female red snapper (*Lutjanus campechanus*) collected from the Gulf of Mexico (Gulf) off Alabama (eastern Gulf) and Louisiana (western Gulf) in April-October of 1999, 2000, 2001, 2009 and 2010. Gonadosomatic index (GSI) values were log_e_ transformed to meet the assumptions of ANOVA. Similar superscript letters indicate no significant difference detected between age groups, according to Tukey’s adjusted least square means test (α = 0.05). SE, standard error.
(DOCX)

S3 Table Analysis of variance (ANOVA) results testing equality of mean gonadosomatic index (GSI) values among four sample groups of female red snapper (*Lutjanus campechanus*) collected from the Gulf of Mexico (Gulf) off Alabama (eastern Gulf) and Louisiana (western Gulf) in 1999, 2000, 2001, 2009 and 2010. Gonadosomatic index values were log_e_ transformed to meet the assumptions of ANOVA. Sample groups correspond with region and year(s) sampled and represented by the following letters: A) EG1, eastern Gulf 1999–2001; B) WG1, western Gulf 1999–2001; C) EG2, eastern Gulf 2009; and D) WG2, western Gulf 2009–2010. For comparisons of mean GSI values with Tukey’s honest significant difference (HSD) post-hoc test, letters separated by commas indicate no significant difference, while letters separated by < or > signs indicate significant differences were detected (α<0.05). M, mean; SD, standard deviation; df, degrees of freedom; SS, sum of squares; MS, mean square; F, F-value; p, p-value.
(DOCX)

S4 Table Monthly mean gonadosomatic index (GSI) values at age (years) with standard deviations for female red snapper (*Lutjanus campechanus*) sampled from the Gulf of Mexico off the Alabama and Louisiana coasts in 1999, 2000, 2001, 2009 and 2010. An asterisk indicates n = 1.
(DOCX)

S5 Table Analyses of variance (ANOVA) testing equality of mean gonadosomatic index (GSI) values among 3 age groups of female red snapper (*Lutjanus campechanus*) collected from the Gulf of Mexico (Gulf) off Alabama and Louisiana in May-August of 1999, 2000, 2001, 2009 and 2010. Age groups correspond with discrete somatic growth rates at age for Gulf red snapper [12] and are discernible by the following letters: A) 2–5 years, B) 6–8 years, and C) ≥9 years. Gonadosomatic index values were log_e_ transformed to meet the assumptions of ANOVA. For comparisons of mean log_e_ GSI values with Tukey’s test, letters separated by commas indicate no significant difference, while letters separated by < or > signs indicate significant differences were detected (α<0.05). M, mean; SD, standard deviation; df, degrees of freedom; SS, sum of squares; MS, mean square; F, F-value; p, p-value.
(DOCX)

S6 Table Analysis of variance (ANOVA) results with standard error (SE) testing for equality of mean monthly gonadosomatic index (GSI) values among 3 age groups (years) of female red snapper, *Lutjanus campechanus*, sampled from the Gulf of Mexico off Alabama and Louisiana in May-August of 1999, 2000, 2001, 2009 and 2010 (n = 1,495). Gonadosomatic index values were log_e_ transformed to meet the assumptions of ANOVA. Similar superscript letters indicate no significant difference detected between age groups, according to Tukey’s adjusted least square means test (α = 0.05).
(DOCX)

S7 Table Analysis of variance (ANOVA) results with standard error (SE) testing equality of mean gonadosomatic index (GSI) values between two age groups of female red snapper (*Lutjanus campechanus*) collected from the Gulf of Mexico (Gulf) off Alabama (eastern Gulf) and Louisiana (western Gulf) in May-August of 1999, 2000, 2001, 2009 and 2010. A = 2–5 year olds; B = ≥6 year olds. Sample groups correspond with regions and years when sampling occurred: EG1 = eastern Gulf 1999–2001, WG1 = western Gulf 1999–2001, EG2 = eastern Gulf 2009, and WG2 = Gulf 2009–2010. Gonadosomatic index values were log_e_ transformed to meet the assumptions of ANOVA. Similar superscript letters indicate no significant difference detected between age groups, according to Tukey’s adjusted least square means test (α = 0.05).
(DOCX)

S8 Table Analysis of variance (ANOVA) results testing equality of mean gonadosomatic index (GSI) values among 4 age groups of female red snapper (*Lutjanus campechanus*) sampled in May, June, July and August. Red snappers were collected from the Gulf of Mexico (Gulf) off Alabama (eastern Gulf) and Louisiana (western Gulf) in 1999, 2000, 2001, 2009 and 2010. Sample groups correspond with region and year(s) sampled and are discernible by the following letters: A) eastern Gulf 1999–2001 (EG1), B) western Gulf 1999–2001 (WG1), C) eastern Gulf 2009 (EG2), and D) western Gulf 2009–2010 (WG2). Gonadosomatic index values were log_e_ transformed to meet the assumptions of ANOVA. For comparisons of mean log_e_ GSI values with Tukey’s honest significant difference (HSD) post-hoc test, letters separated by commas indicate no significant difference, while letters separated by < or > signs indicate significant differences were detected (α<0.05). M, mean; SD, standard deviation; df, degrees of freedom; SS, sum of squares; MS, mean square; F, F-value; p, p-value.
(DOCX)

S9 Table Analysis of variance (ANOVA) results testing equality of mean gonadosomatic index (GSI) values among 4 age groups of female red snapper (*Lutjanus campechanus*) sampled in July. Red snapper weres collected from the Gulf of Mexico (Gulf) off Alabama (eastern Gulf) and Louisiana (western Gulf) in 1999, 2000, 2001, 2009 and 2010. Sample groups correspond with region and year(s) sampled and are discernible by the following letters: A) eastern Gulf 1999–2001 (EG1), B) western Gulf 1999–2001 (WG1), C) eastern Gulf 2009 (EG2), and D) western Gulf 2009–2010 (WG2). Gonadosomatic index values were log_e_ transformed to meet the assumptions of ANOVA. For comparisons of mean log_e_ GSI values with Tukey’s honest significant difference post-hoc test, letters separated by commas indicate no significant difference, while letters separated by < or > signs indicate significant differences were detected (α<0.05). M, mean; SD, standard deviation; df, degrees of freedom; SS, sum of squares; MS, mean square; F, F-value; p, p-value.
(DOCX)

S10 Table Analysis of variance (ANOVA) results testing equality of mean gonadosomatic index (GSI) values between two age groups of female red snapper (*Lutjanus campechanus*) collected from the Gulf of Mexico (Gulf) off Alabama (eastern Gulf) and Louisiana (western Gulf) in July of 1999, 2000, 2001, 2009 and 2010. Age groups and are discernible by the following letters: A) 6–8 year olds; B) ≥9 year olds. Sample groups correspond with region and year(s) sampled and are represented by the following: EG1, eastern Gulf 1999–2001; WG1, western Gulf 1999–2001; EG2, eastern Gulf 2009; WG2, Gulf 2009–2010. Gonadosomatic index (GSI) values were log_e_ transformed to meet the assumptions of ANOVA. Similar superscript letters indicate no significant difference detected between age groups, according to Tukey’s adjusted least square means test (α = 0.05). M, mean; SE, standard error.
(DOCX)

S11 Table Percent maturity at fork length (millimeters) among female red snapper (*Lutjanus campechanus*) collected from the Gulf of Mexico (Gulf) off Alabama (eastern Gulf) and Louisiana (western Gulf) in 1999, 2000 and 2001, as described by Jackson et al. [11]. FL, fork length.
(DOCX)

S12 Table Spawning frequency (SF) estimates among three age groups of female red snapper (*Lutjanus campechanus*) sampled from the Gulf of Mexico off Alabama (eastern Gulf) in 2009 and Louisiana (western Gulf) in 2009 and 2010. EG2, eastern Gulf 2009; WG2, western Gulf 2009–2010; SF_H_, spawning frequency estimate based on the hydrated oocyte method; SF_POF_, spawning frequency estimate based on the POF method; SF_TC_, spawning frequency estimate based on the time-calibrated method. *Unusually small number of individuals with POF (n = 3) but high number with H+ (n = 31) for 2–5 year olds sampled from the east. ^Small total sample size (n = 5) and no individuals with POF found led to low SF_TC_ estimate.
(DOCX)

S13 Table Chi-Square test of independence and Fisher’s exact test p-values for spawning frequency estimates compared among female red snapper (*Lutjanus campechanus*) sampled from Gulf of Mexico off Alabama (eastern Gulf) and Louisiana (western Gulf) in 2009 and 2010. Spawning frequency was estimated using the hydrated oocyte method, the post-ovulatory follicle method, and the time-calibrated method. EG2, eastern Gulf 2009; WG2, western Gulf 2009–2010. *Insufficient sample size for Chi Square test of independence, so Fisher's Exact Test was used. ^Insufficient sample size for Chi Square and Fisher's exact test.
(DOCX)

S14 Table Mann-Whitney-Wilcoxon results comparing nonparametric batch fecundity means among 3 age groups of female red snapper (*Lutjanus campechanus*). Red snapper were sampled from the Gulf of Mexico off the Alabama and Louisiana coastlines in 1999, 2000, 2001 and 2009. EG1, eastern Gulf 1999–2001; WG1, western Gulf 1999–2001; EG2, eastern Gulf 2009; WG2, western Gulf 2009; n, sample size; min, minimum; max, maximum; SE, standard error. * No significant difference found when similar ages were compared (4 and 5 year olds).
(DOCX)

S15 Table Mann-Whitney-Wilcoxon results comparing nonparametric mean batch fecundity among 4 and 5 year old female red snappers. Red snapper were sampled from the Gulf of Mexico off Alabama and Louisiana in 1999, 2000, 2001 and 2009. EG1, eastern Gulf 1999–2001; WG1, western Gulf 1999–2001; EG2, eastern Gulf 2009; WG2, western Gulf 2009; n, sample size; min, minimum; max, maximum; SE, standard error. Similar superscripted letters indicate no significant difference among means.
(DOCX)

S1 Fig Box plot of mean gonadosomatic index (GSI) values among 2 to 5 year old female red snapper (*Lutjanus campechanus*) collected from the Gulf of Mexico off Alabama (eastern Gulf) and Louisiana (western Gulf) in 1999, 2000, 2001, 2009 and 2010. Gonadosomatic index values were log_e_ transformed (LGSI) to meet the assumptions of analysis of variance (ANOVA). Sample groups correspond with region and year(s) sampled: EG1, eastern Gulf 1999–2001; WG1, western Gulf 1999–2001; EG2, eastern Gulf 2009; WG2, western Gulf 2009–2010.
(DOCX)

S2 Fig Box plot of mean gonadosomatic index (GSI) values among female red snapper (*Lutjanus campechanus*) ≥6 years old collected from the Gulf of Mexico off Alabama (eastern Gulf) and Louisiana (western Gulf) in 1999, 2000, 2001, 2009 and 2010. Gonadosomatic index values were log_e_ transformed (LGSI) to meet the assumptions of analysis of variance (ANOVA). Sample groups correspond with region and year(s) sampled: EG1, eastern Gulf 1999–2001; WG1, western Gulf 1999–2001; EG2, eastern Gulf 2009; WG2, western Gulf 2009–2010.
(DOCX)

S3 Fig Gonadosomatic index (GSI) values plotted against fork length (millimeters) for female red snapper (*Lutjanus campechanus*) sampled from the Gulf of Mexico off Alabama and Louisiana. Sampling occurred in May-August of 1999, 2000, 2001, 2009 and 2010 (n = 1,501).
(DOCX)

S4 Fig Gonadosomatic index (GSI) values at age (years) for female red snapper (*Lutjanus campechanus*) sampled from the Gulf of Mexico off Alabama and Louisiana. Sampling occurred in May through August of 1999, 2000, 2001, 2009 and 2010 (n = 1,502).
(DOCX)

S5 Fig Regression relationship between log_e_ transformations of gonadosomatic index (GSI) values and fork length (millimeters) for female red snapper (*Lutjanus campechanus*) sampled from the Gulf of Mexico. Sampling occurred off Alabama and Louisiana in May-August of 1999, 2000, 2001, 2009 and 2010 (n = 1,496).
(DOCX)

S6 Fig Regression relationship between log_e_ transformations of gonadosomatic index (GSI) values and age (years) for female red snapper (*Lutjanus campechanus*) sampled from the northern Gulf of Mexico. Sampling occurred off Alabama and Louisiana in May-August of 1999, 2000, 2001, 2009 and 2010 (n = 1,497).
(DOCX)

S7 Fig Percent maturity at age among female red snapper (*Lutjanus campechanus*) collected exclusively from artificial reef habitats in the western Gulf of Mexico (off Louisiana) in 1999, 2000, 2001, 2009 and 2010. Sample groups correspond with region and year(s) sampled: WG1, western Gulf 1999–2001; WG2, western Gulf 2009–2010.
(DOCX)

S8 Fig Mean batch fecundity (BF) at age for female red snapper (*Lutjanus campechanus*) collected from the Gulf of Mexico (Gulf) off Alabama (eastern Gulf) and Louisiana (western Gulf) in 1999, 2000, 2001 and 2009. Sample groups correspond with region and year(s) sampled: EG1, eastern Gulf 1999–2001; WG1, western Gulf 1999–2001; EG2, eastern Gulf 2009; WG2, western Gulf 2009.
(DOCX)

S9 Fig Box plot of mean batch fecundity (BF) among three age groups of female red snapper (*Lutjanus campechanus*) sampled from the Gulf of Mexico off Alabama and Louisiana in 1999, 2000, 2001 and 2009. A, 2–5 year olds; B, 6–8 year olds; C, ≥9 year olds.
(DOCX)

S10 Fig Box plot of mean batch fecundity (BF) among 2–5 year old female red snappers (*Lutjanus campechanus*) sampled from the Gulf of Mexico off Alabama and Louisiana in 1999, 2000, 2001 and 2009. Sample groups correspond with region and year(s) sampled: EG1, eastern Gulf 1999–2001; WG1, western Gulf 1999–2001; EG2, eastern Gulf 2009; WG2, western Gulf 2009.
(DOCX)

S11 Fig Box plot of mean batch fecundity (BF) among 6–8 year old female red snappers (*Lutjanus campechanus*) collected from the Gulf of Mexico off Alabama and Louisiana in 1999–2001. Sample groups correspond with region and year(s) sampled: EG1, eastern Gulf 1999–2001; WG1, western Gulf 1999–2001.
(DOCX)
