# Supplementary material for: Temporal and spatial comparisons of the reproductive biology of northern Gulf of Mexico (USA) red snapper (Lutjanus campechanus) collected a decade apart
Source: PLoS One. 2017 Mar 29;12(3):e0172360. doi: 10.1371/journal.pone.0172360 (PMC5371290; doi:10.1371/journal.pone.0172360)
Supplement: S1 Table — (DOCX) [file pone.0172360.s001.docx]

| Month | n | Min | Max | Mean ± SE |
| --- | --- | --- | --- | --- |
| April | 52 | 0.15 | 3.99 | 0.77 ± 0.13 |
| May | 183 | 0.13 | 9.3 | 2.36 ± 0.15 |
| June | 426 | 0.15 | 15.11 | 2.62 ± 0.11 |
| July | 678 | 0.05 | 10.65 | 2.33 ± 0.07 |
| August | 281 | 0.13 | 7.13 | 1.41 ± 0.08 |
| September | 140 | 0.14 | 3.95 | 0.84 ± 0.07 |
| October | 10 | 0.32 | 2.01 | 0.63 ± 0.16 |
