# Supplementary material for: Temporal and spatial comparisons of the reproductive biology of northern Gulf of Mexico (USA) red snapper (Lutjanus campechanus) collected a decade apart
Source: PLoS One. 2017 Mar 29;12(3):e0172360. doi: 10.1371/journal.pone.0172360 (PMC5371290; doi:10.1371/journal.pone.0172360)
Supplement: S2 Table — Gonadosomatic index (GSI) values were loge transformed to meet the assumptions of ANOVA. Similar superscript letters indicate no significant difference detected between age groups, according to Tukey’s adjusted least square means test (α = 0.05). SE, standard error. (DOCX) [file pone.0172360.s002.docx]

| **April** | n | Min | Max | Mean ± SE |
| --- | --- | --- | --- | --- |
| EG1 | - | - | - | - |
| WG1 | 49 | 0.15 | 3.99 | 0.77 ± 0.14 |
| EG2 | - | - | - | - |
| WG2 | 3* | 0.62 | 1.1 | 0.80 ± 0.15 |
| **May** | n | Min | Max | Mean ± SE |
| EG1 | 75 | 0.18 | 9.3 | 2.73 ± 0.23^A^ |
| WG1 | 108 | 0.13 | 8.87 | 2.10 ± 0.20^B^ |
| EG2 | - | - | - | - |
| WG2 | - | - | - | - |
| **June** | n | Min | Max | Mean ± SE |
| EG1 | 139 | 0.28 | 9.57 | 3.23 ± 0.19^A^ |
| WG1 | 141 | 0.25 | 15.11 | 2.79 ± 0.20^A^ |
| EG2 | 93 | 0.3 | 15.03 | 2.15 ± 0.22^B^ |
| WG2 | 53 | 0.15 | 3.37 | 1.35 ± 0.13^B^ |
| **July** | n | Min | Max | Mean ± SE |
| EG1 | 373 | 0.11 | 10.65 | 3.02 ± 0.10^A^ |
| WG1 | 177 | 0.05 | 6.56 | 1.54 ± 0.10^B^ |
| EG2 | - | - | - | - |
| WG2 | 128 | 0.14 | 4.75 | 1.40 ± 0.10^B^ |
| **August** | n | Min | Max | Mean ± SE |
| EG1 | 168 | 0.16 | 6.89 | 1.69 ± 0.10^A^ |
| WG1 | 90 | 0.13 | 7.13 | 1.07 ± 0.16^B^ |
| EG2 | - | - | - | - |
| WG2 | 23 | 0.2 | 2.96 | 0.64 ± 0.14^B^ |
| **September** | n | Min | Max | Mean ± SE |
| EG1 | 59 | 0.47 | 3.95 | 1.46 ± 0.12^A^ |
| WG1 | 81 | 0.14 | 1.5 | 0.39 ± 0.02^B^ |
| EG2 | - | - | - | - |
| WG2 | - | - | - | - |
| **October** | n | Min | Max | Mean ± SE |
| EG1 | 4* | 0.33 | 0.8 | 0.53 ± 0.10 |
| WG1 | - | - | - | - |
| EG2 | - | - | - | - |
| WG2 | 6* | 0.32 | 2.01 | 0.69 ± 0.27 |
