# Supplementary material for: Temporal and spatial comparisons of the reproductive biology of northern Gulf of Mexico (USA) red snapper (Lutjanus campechanus) collected a decade apart
Source: PLoS One. 2017 Mar 29;12(3):e0172360. doi: 10.1371/journal.pone.0172360 (PMC5371290; doi:10.1371/journal.pone.0172360)
Supplement: S3 Table — Gonadosomatic index values were loge transformed to meet the assumptions of ANOVA. Sample groups correspond with region and year(s) sampled and represented by the following letters: A) EG1, eastern Gulf 1999–2001; B) WG1, western Gulf 1999–2001; C) EG2, eastern Gulf 2009; and D) WG2, western Gulf 2009–2010. For comparisons of mean GSI values with Tukey’s honest significant difference (HSD) post-hoc test, letters separated by commas indicate no significant difference, while letters separated by < or > signs indicate significant differences were detected (α<0.05). M, mean; SD, standard deviation; df, degrees of freedom; SS, sum of squares; MS, mean square; F, F-value; p, p-value. (DOCX) [file pone.0172360.s003.docx]

|  | A | |  | B | |  | C | |  | D | |  | ANOVA | | | | |  |
| --- | --- | --- | --- | --- | --- | --- | --- | --- | --- | --- | --- | --- | --- | --- | --- | --- | --- | --- |
| Month | M | SD |  | M | SD |  | M | SD |  | M | SD |  | df | SS | MS | F | p | Tukey's HSD |
| May | 2.73 | 2.02 |  | 2.10 | 2.09 |  | - | - |  | - | - |  | 1 | 13.14 | 13.14 | 12.57 | 0.0005 | A > B |
| June | 3.23 | 2.23 |  | 2.79 | 2.32 |  | 2.15 | 1.68 |  | 1.35 | 0.95 |  | 3 | 39.85 | 13.28 | 20.40 | <0.0001 | A,B > C,D |
| July | 3.02 | 1.95 |  | 1.54 | 1.40 |  | - | - |  | 1.40 | 1.14 |  | 2 | 112.02 | 56.01 | 84.74 | <0.0001 | A > B,D |
| August | 1.69 | 1.31 |  | 1.07 | 1.03 |  | - | - |  | 0.64 | 0.68 |  | 2 | 61.76 | 30.88 | 45.78 | <0.0001 | A > B,D |
