# Supplementary material for: Temporal and spatial comparisons of the reproductive biology of northern Gulf of Mexico (USA) red snapper (Lutjanus campechanus) collected a decade apart
Source: PLoS One. 2017 Mar 29;12(3):e0172360. doi: 10.1371/journal.pone.0172360 (PMC5371290; doi:10.1371/journal.pone.0172360)
Supplement: S4 Table — An asterisk indicates n = 1. (DOCX) [file pone.0172360.s004.docx]

| Age | n | April | May | June | July | August | September | October |
| --- | --- | --- | --- | --- | --- | --- | --- | --- |
| 2 | 79 | - | - | 1.62 ± 1.21 | 0.60 ± 0.40 | 0.78 ± 0.69 | 1.26 ± 0.71 | 0.44 ± 0.12 |
| 3 | 416 | 0.33 ± 0.23 | 2.02 ±1.89 | 1.74 ± 1.61 | 1.29 ± 1.09 | 1.10 ± 1.19 | 0.61 ± 0.73 | 0.32* |
| 4 | 537 | 0.27 ± 0.13 | 1.81 ± 1.99 | 2.45 ± 1.98 | 2.04 ± 1.95 | 1.11 ± .082 | 0.89 ± 0.74 | 0.54 ± 0.22 |
| 5 | 322 | 0.46 ± 0.27 | 1.72 ± 1.46 | 2.11 ± 1.66 | 2.10 ± 1.86 | 1.36 ± 1.14 | 1.90 ± 0.92 | 0.46* |
| 6 | 116 | 1.69 ± 0.75 | 3.07 ± 1.74 | 3.79 ± 1.96 | 2.87 ± 1.58 | 3.08 ± 2.00 | 0.88* | - |
| 7 | 82 | 2.88 ± 0.67 | 3.72 ± 1.61 | 3.82 ± 1.59 | 2.98 ± 1.07 | 4.25 ± 1.62 | - | - |
| 8 | 42 | 1.82 ± 0.63 | 4.74 ± 2.41 | 3.93 ± 1.64 | 3.64 ± 2.01 | 3.87 ± 0.86 | - | - |
| ≥9 | 130 | 2.72 ± 1.80 | 5.44 ± 2.04 | 5.71 ± 3.06 | 3.88 ± 1.48 | 4.43 ± 0.80 | 1.61* | 2.01* |
| Total/Mean | 1724 | 0.77 ± 0.92 | 2.34 ± 2.10 | 2.62 ± 2.15 | 2.32 ± 1.85 | 1.38 ± 1.36 | 0.84 ± 0.80 | 0.63 ± 0.51 |
