# Supplementary material for: Temporal and spatial comparisons of the reproductive biology of northern Gulf of Mexico (USA) red snapper (Lutjanus campechanus) collected a decade apart
Source: PLoS One. 2017 Mar 29;12(3):e0172360. doi: 10.1371/journal.pone.0172360 (PMC5371290; doi:10.1371/journal.pone.0172360)
Supplement: S5 Table — Age groups correspond with discrete somatic growth rates at age for Gulf red snapper [12] and are discernible by the following letters: A) 2–5 years, B) 6–8 years, and C) ≥9 years. Gonadosomatic index values were loge transformed to meet the assumptions of ANOVA. For comparisons of mean loge GSI values with Tukey’s test, letters separated by commas indicate no significant difference, while letters separated by < or > signs indicate significant differences were detected (α<0.05). M, mean; SD, standard deviation; df, degrees of freedom; SS, sum of squares; MS, mean square; F, F-value; p, p-value. (DOCX) [file pone.0172360.s005.docx]

|  | A | |  | B | |  | C | |  | ANOVA | | | | |  |
| --- | --- | --- | --- | --- | --- | --- | --- | --- | --- | --- | --- | --- | --- | --- | --- |
| Month | M | SD |  | M | SD |  | M | SD |  | df | SS | MS | F | p | Tukey's HSD |
| May | 1.83 | 1.71 |  | 3.76 | 1.92 |  | 5.44 | 2.04 |  | 2 | 40.58 | 20.29 | 22.32 | <0.0001 | A < B,C |
| June | 2.19 | 1.80 |  | 3.81 | 1.73 |  | 5.70 | 3.07 |  | 2 | 53.18 | 26.59 | 47.19 | <0.0001 | A < B,C |
| July | 1.86 | 1.78 |  | 3.00 | 1.55 |  | 3.87 | 1.49 |  | 2 | 115.84 | 57.92 | 88.21 | <0.0001 | A < B < C |
| August | 1.09 | 0.94 |  | 3.89 | 1.50 |  | 4.43 | 0.80 |  | 2 | 58.52 | 29.26 | 42.61 | <0.0001 | A < B,C |
