# Supplementary material for: Temporal and spatial comparisons of the reproductive biology of northern Gulf of Mexico (USA) red snapper (Lutjanus campechanus) collected a decade apart
Source: PLoS One. 2017 Mar 29;12(3):e0172360. doi: 10.1371/journal.pone.0172360 (PMC5371290; doi:10.1371/journal.pone.0172360)
Supplement: S9 Table — Red snapper weres collected from the Gulf of Mexico (Gulf) off Alabama (eastern Gulf) and Louisiana (western Gulf) in 1999, 2000, 2001, 2009 and 2010. Sample groups correspond with region and year(s) sampled and are discernible by the following letters: A) eastern Gulf 1999–2001 (EG1), B) western Gulf 1999–2001 (WG1), C) eastern Gulf 2009 (EG2), and D) western Gulf 2009–2010 (WG2). Gonadosomatic index values were loge transformed to meet the assumptions of ANOVA. For comparisons of mean loge GSI values with Tukey’s honest significant difference post-hoc test, letters separated by commas indicate no significant difference, while letters separated by < or > signs indicate significant differences were detected (α<0.05). M, mean; SD, standard deviation; df, degrees of freedom; SS, sum of squares; MS, mean square; F, F-value; p, p-value. (DOCX) [file pone.0172360.s009.docx]

|  | A | |  | B | |  | C | |  | D | | |  | ANOVA | | | | |  |
| --- | --- | --- | --- | --- | --- | --- | --- | --- | --- | --- | --- | --- | --- | --- | --- | --- | --- | --- | --- |
| Age Group | M | SD |  | M | SD |  | M | SD |  | M | SD | |  | df | SS | MS | F | p | Tukey's HSD |
| 6-8 years | 3.49 | 1.58 |  | 2.49 | 1.32 |  | - | - |  | 2.30 | | 1.23 |  | 2 | 6.22 | 3.11 | 11.15 | <0.0001 | A > B,D |
| ≥9 years | 4.00 | 1.52 |  | 3.57 | 1.44 |  | - | - |  | - | | - |  | 1 | 0.24 | 0.24 | 1.88 | 0.1747 | A,B |
