# Supplementary material for: Temporal and spatial comparisons of the reproductive biology of northern Gulf of Mexico (USA) red snapper (Lutjanus campechanus) collected a decade apart
Source: PLoS One. 2017 Mar 29;12(3):e0172360. doi: 10.1371/journal.pone.0172360 (PMC5371290; doi:10.1371/journal.pone.0172360)
Supplement: S10 Table — Age groups and are discernible by the following letters: A) 6–8 year olds; B) ≥9 year olds. Sample groups correspond with region and year(s) sampled and are represented by the following: EG1, eastern Gulf 1999–2001; WG1, western Gulf 1999–2001; EG2, eastern Gulf 2009; WG2, Gulf 2009–2010. Gonadosomatic index (GSI) values were loge transformed to meet the assumptions of ANOVA. Similar superscript letters indicate no significant difference detected between age groups, according to Tukey’s adjusted least square means test (α = 0.05). M, mean; SE, standard error. (DOCX) [file pone.0172360.s010.docx]

| **A** | n | Min | Max | Mean ± SE |
| --- | --- | --- | --- | --- |
| EG1 | 69 | 1.14 | 8.61 | 3.49 ± 0.19^A^ |
| WG1 | 30 | 0.51 | 5.4 | 2.49 ± 0.24^B^ |
| EG2 | - | - | - | - |
| WG2 | 26 | 0.63 | 4.62 | 2.30 ± 0.24^B^ |
| **B** | n | Min | Max | Mean ± SE |
| EG1 | 60 | 1.76 | 9.57 | 4.00 ± 1.52^A^ |
| WG1 | 20 | 1.38 | 6.56 | 3.57 ± 1.44^A^ |
| EG2 | - | - | - | - |
| WG2 | 3 | 2.41 | 4.36 | 3.40 ± 0.98 |
