# Supplementary material for: Temporal and spatial comparisons of the reproductive biology of northern Gulf of Mexico (USA) red snapper (Lutjanus campechanus) collected a decade apart
Source: PLoS One. 2017 Mar 29;12(3):e0172360. doi: 10.1371/journal.pone.0172360 (PMC5371290; doi:10.1371/journal.pone.0172360)
Supplement: S11 Table — FL, fork length. (DOCX) [file pone.0172360.s011.docx]

|  | | |  |
| --- | --- | --- | --- |
| **FL** | **n** | **n mature** | **% mature** |
| 250 | 13 | 10 | 77 |
| 300 | 77 | 64 | 83 |
| 350 | 111 | 97 | 87 |
| 400 | 189 | 166 | 88 |
| 450 | 240 | 206 | 86 |
| 500 | 142 | 123 | 87 |
| 550 | 81 | 75 | 93 |
| 600 | 47 | 46 | 98 |
