# Supplementary material for: Temporal and spatial comparisons of the reproductive biology of northern Gulf of Mexico (USA) red snapper (Lutjanus campechanus) collected a decade apart
Source: PLoS One. 2017 Mar 29;12(3):e0172360. doi: 10.1371/journal.pone.0172360 (PMC5371290; doi:10.1371/journal.pone.0172360)
Supplement: S12 Table — EG2, eastern Gulf 2009; WG2, western Gulf 2009–2010; SFH, spawning frequency estimate based on the hydrated oocyte method; SFPOF, spawning frequency estimate based on the POF method; SFTC, spawning frequency estimate based on the time-calibrated method. *Unusually small number of individuals with POF (n = 3) but high number with H+ (n = 31) for 2–5 year olds sampled from the east. ^Small total sample size (n = 5) and no individuals with POF found led to low SFTC estimate. (DOCX) [file pone.0172360.s012.docx]

| 2-5 years | n | Min | Max | Mean ± SE |
| --- | --- | --- | --- | --- |
| EG1 | 121 | 693 | 1772050 | 204219 ± 20869^A^* |
| WG1 | 42 | 1412 | 1243114 | 189707 ± 49940^B^ |
| EG2 | 22 | 45817 | 615702 | 279247 ± 40532^C^* |
| WG2 | 11 | 4631 | 316514 | 55015 ± 27282^B^ |
| 6-8 years | n | Min | Max | Mean ± SE |
| EG1 | 17 | 107067 | 1564691 | 812470 ± 115731^A^ |
| WG1 | 21 | 72656 | 1701582 | 693996 ± 94696^A^ |
| EG2 | - | - | - | - |
| WG2 | 2 | 326734 | 945114 | 635924 ± 309190 |
| ≥9 years | n | Min | Max | Mean ± SE |
| EG1 | 3 | 1351074 | 1903091 | 1559037 ± 173274 |
| WG1 | 12 | 512443 | 2236575 | 1578306 ± 151956 |
| EG2 | - | - | - | - |
| WG2 | - | - | - | - |
