# Supplementary material for: Temporal and spatial comparisons of the reproductive biology of northern Gulf of Mexico (USA) red snapper (Lutjanus campechanus) collected a decade apart
Source: PLoS One. 2017 Mar 29;12(3):e0172360. doi: 10.1371/journal.pone.0172360 (PMC5371290; doi:10.1371/journal.pone.0172360)
Supplement: S13 Table — Spawning frequency was estimated using the hydrated oocyte method, the post-ovulatory follicle method, and the time-calibrated method. EG2, eastern Gulf 2009; WG2, western Gulf 2009–2010. *Insufficient sample size for Chi Square test of independence, so Fisher's Exact Test was used. ^Insufficient sample size for Chi Square and Fisher's exact test. (DOCX) [file pone.0172360.s013.docx]

| 4-5 years | n | Min | Max | Mean ± SE |
| --- | --- | --- | --- | --- |
| EG1 | 83 | 3310 | 1772050 | 257101 ± 28256^A^ |
| WG1 | 21 | 4567 | 1243114 | 328455 ± 88266^AB^ |
| EG2 | 22 | 45817 | 615702 | 279247 ± 40532^A^ |
| WG2 | 9 | 7556 | 316514 | 64239 ± 32809^B^ |
