# Supplementary figures and images for: Temporal and spatial comparisons of the reproductive biology of northern Gulf of Mexico (USA) red snapper (Lutjanus campechanus) collected a decade apart
Source: PLoS One. 2017 Mar 29;12(3):e0172360. doi: 10.1371/journal.pone.0172360 (PMC5371290; doi:10.1371/journal.pone.0172360)

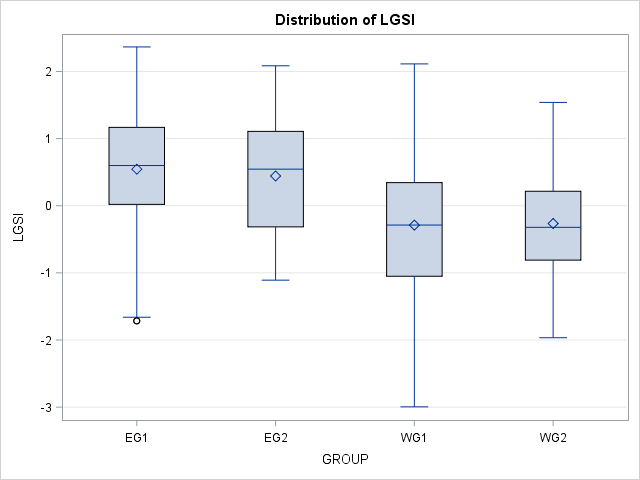

Supplement: S1 Fig — Gonadosomatic index values were loge transformed (LGSI) to meet the assumptions of analysis of variance (ANOVA). Sample groups correspond with region and year(s) sampled: EG1, eastern Gulf 1999–2001; WG1, western Gulf 1999–2001; EG2, eastern Gulf 2009; WG2, western Gulf 2009–2010. (DOCX) [file pone.0172360.s016.docx]

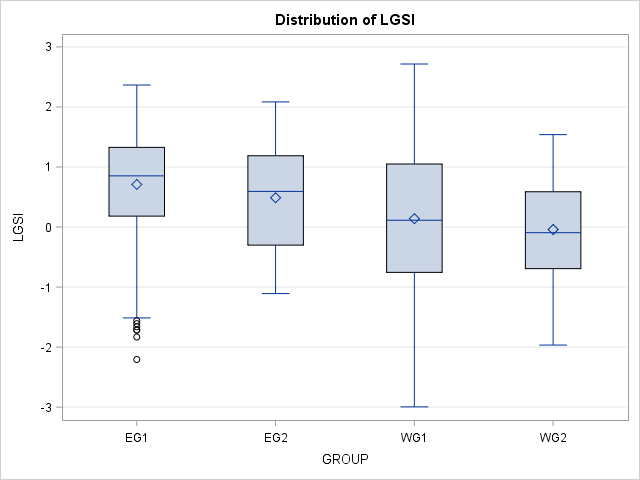

Supplement: S2 Fig — Gonadosomatic index values were loge transformed (LGSI) to meet the assumptions of analysis of variance (ANOVA). Sample groups correspond with region and year(s) sampled: EG1, eastern Gulf 1999–2001; WG1, western Gulf 1999–2001; EG2, eastern Gulf 2009; WG2, western Gulf 2009–2010. (DOCX) [file pone.0172360.s017.docx]

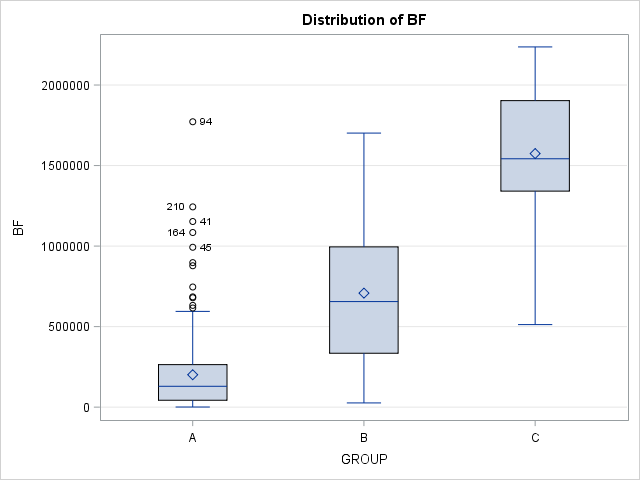

Supplement: S9 Fig — A, 2–5 year olds; B, 6–8 year olds; C, ≥9 year olds. (DOCX) [file pone.0172360.s024.docx]

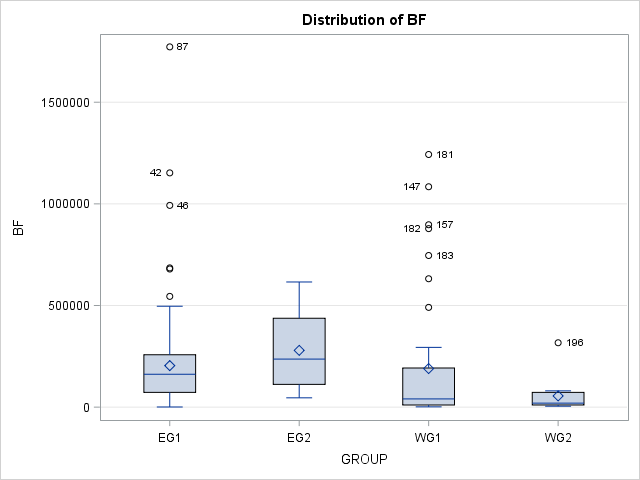

Supplement: S10 Fig — Sample groups correspond with region and year(s) sampled: EG1, eastern Gulf 1999–2001; WG1, western Gulf 1999–2001; EG2, eastern Gulf 2009; WG2, western Gulf 2009. (DOCX) [file pone.0172360.s025.docx]

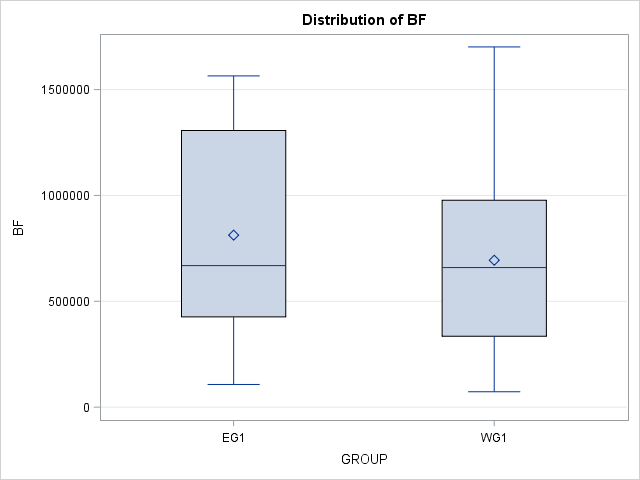

Supplement: S11 Fig — Sample groups correspond with region and year(s) sampled: EG1, eastern Gulf 1999–2001; WG1, western Gulf 1999–2001. (DOCX) [file pone.0172360.s026.docx]
