# Supplementary material for: Temporal and spatial comparisons of the reproductive biology of northern Gulf of Mexico (USA) red snapper (Lutjanus campechanus) collected a decade apart
Source: PLoS One. 2017 Mar 29;12(3):e0172360. doi: 10.1371/journal.pone.0172360 (PMC5371290; doi:10.1371/journal.pone.0172360)
Supplement: Supplementary file 15 [file pone.0172360.s018.docx]

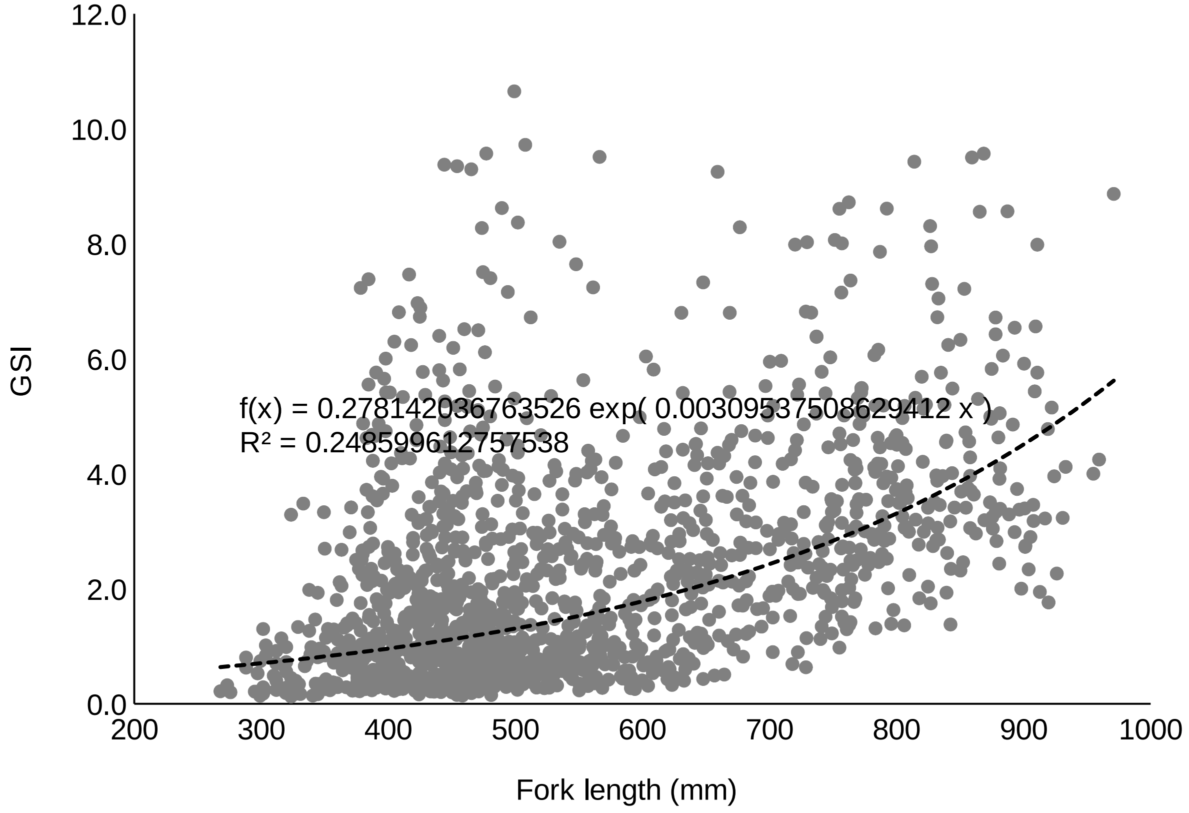

12.0
10.0
8.0
6.0
4.0
2.0
0.0
GSI
f(x) = 0.278142036763526 exp(0.00309537508629412 x)
R² = 0.248599612757538
200
300
400
500
600
700
800
900
1000
Fork length (mm)
