# Supplementary material for: Temporal and spatial comparisons of the reproductive biology of northern Gulf of Mexico (USA) red snapper (Lutjanus campechanus) collected a decade apart
Source: PLoS One. 2017 Mar 29;12(3):e0172360. doi: 10.1371/journal.pone.0172360 (PMC5371290; doi:10.1371/journal.pone.0172360)
Supplement: Supplementary file 16 [file pone.0172360.s019.docx]

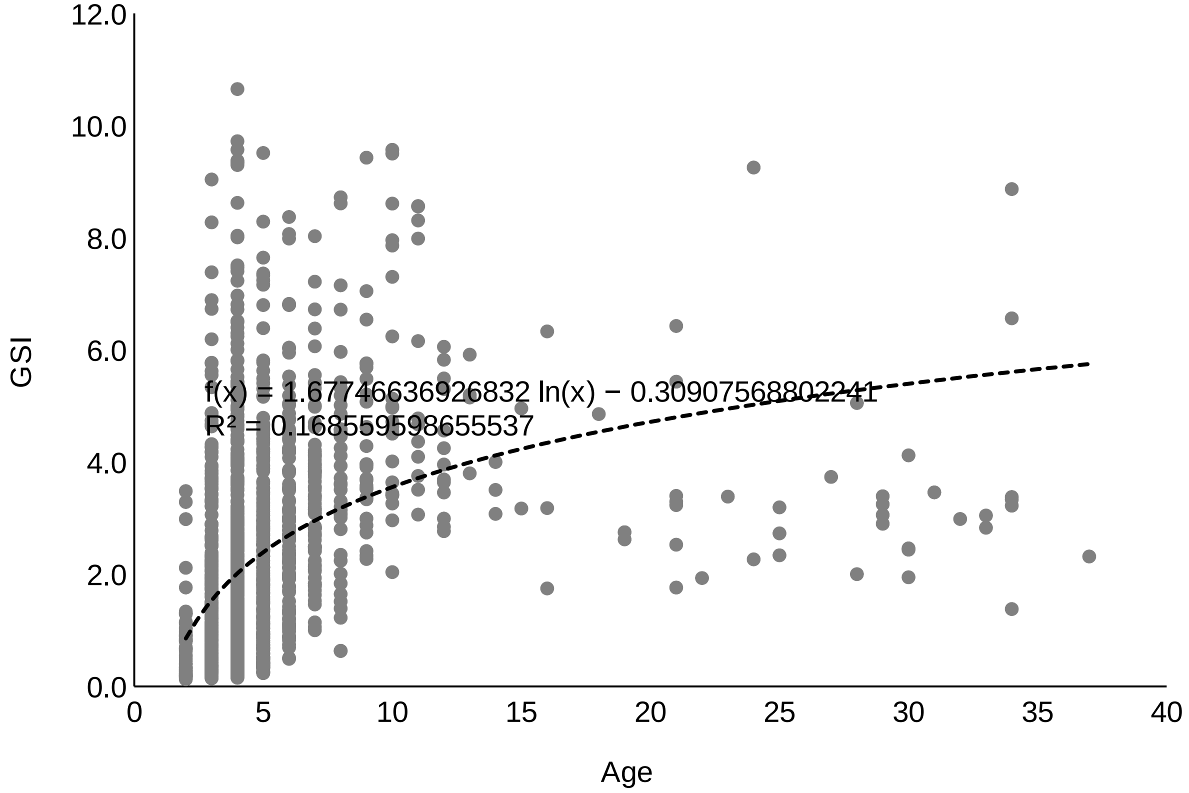
12.0
10.0
8.0
6.0
4.0
2.0
0.0
GSI
0
5
10
15
20
25
30
35
40
Age
f(x) = 1.67746636926832 ln(x) − 0.30907568802241
R² = 0.168559598655537
