# Supplementary material for: Temporal and spatial comparisons of the reproductive biology of northern Gulf of Mexico (USA) red snapper (Lutjanus campechanus) collected a decade apart
Source: PLoS One. 2017 Mar 29;12(3):e0172360. doi: 10.1371/journal.pone.0172360 (PMC5371290; doi:10.1371/journal.pone.0172360)
Supplement: Supplementary file 17 [file pone.0172360.s020.docx]

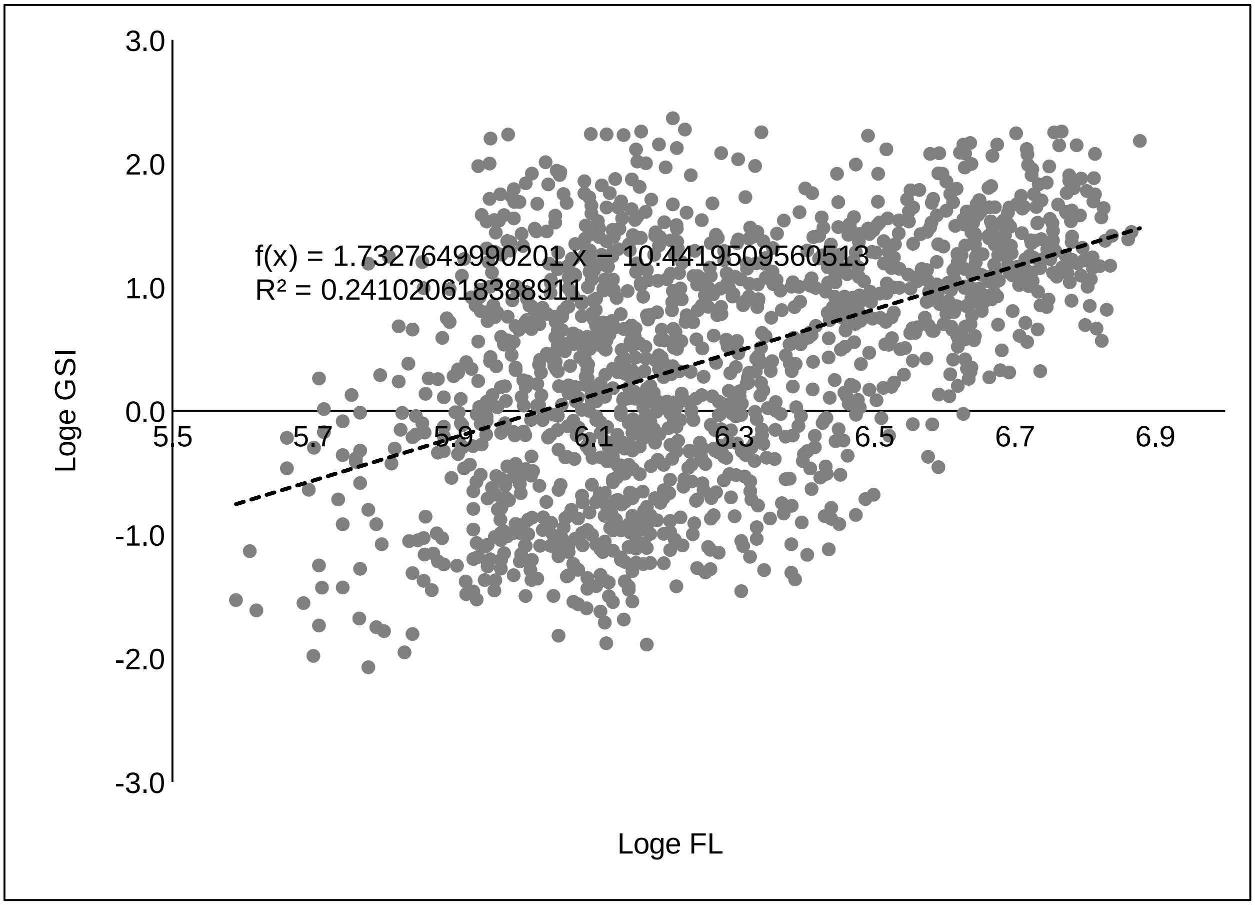

f(x) = 1.7327649990201 x − 10.4419509560513
R² = 0.241020618388911
3.0
2.0
1.0
0.0
-1.0
-2.0
-3.0
5.5
5.7
5.9
6.1
6.3
6.5
6.7
6.9
Loge GSI
Loge FL
