# Supplementary material for: Temporal and spatial comparisons of the reproductive biology of northern Gulf of Mexico (USA) red snapper (Lutjanus campechanus) collected a decade apart
Source: PLoS One. 2017 Mar 29;12(3):e0172360. doi: 10.1371/journal.pone.0172360 (PMC5371290; doi:10.1371/journal.pone.0172360)
Supplement: Supplementary file 18 [file pone.0172360.s021.docx]

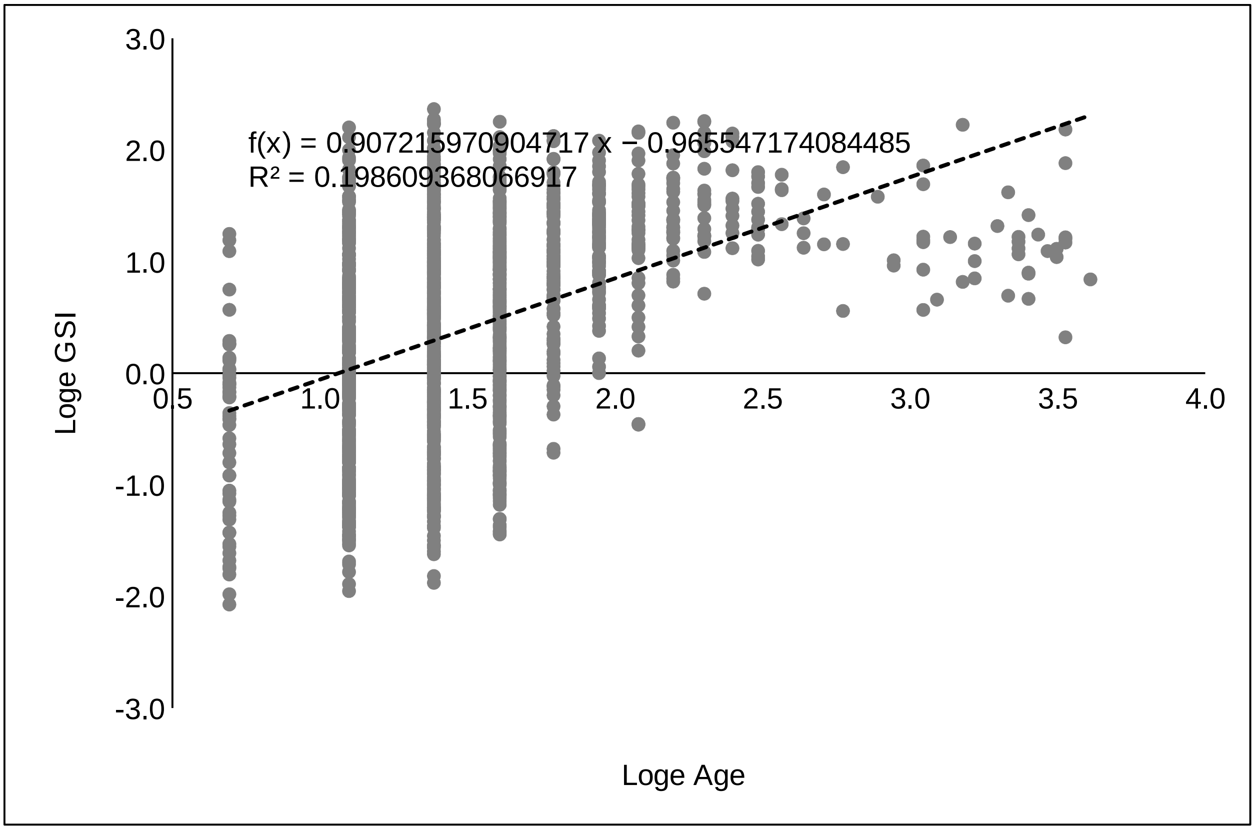

f(x) = 0.907215970904717 x − 0.965547174084485
R² = 0.198609368066917
3.0
2.0
1.0
0.0
-1.0
-2.0
-3.0
0.5
1.0
1.5
2.0
2.5
3.0
3.5
4.0
Loge GSI
Loge Age
