# Supplementary material for: Temporal and spatial comparisons of the reproductive biology of northern Gulf of Mexico (USA) red snapper (Lutjanus campechanus) collected a decade apart
Source: PLoS One. 2017 Mar 29;12(3):e0172360. doi: 10.1371/journal.pone.0172360 (PMC5371290; doi:10.1371/journal.pone.0172360)
Supplement: Supplementary file 19 [file pone.0172360.s022.docx]

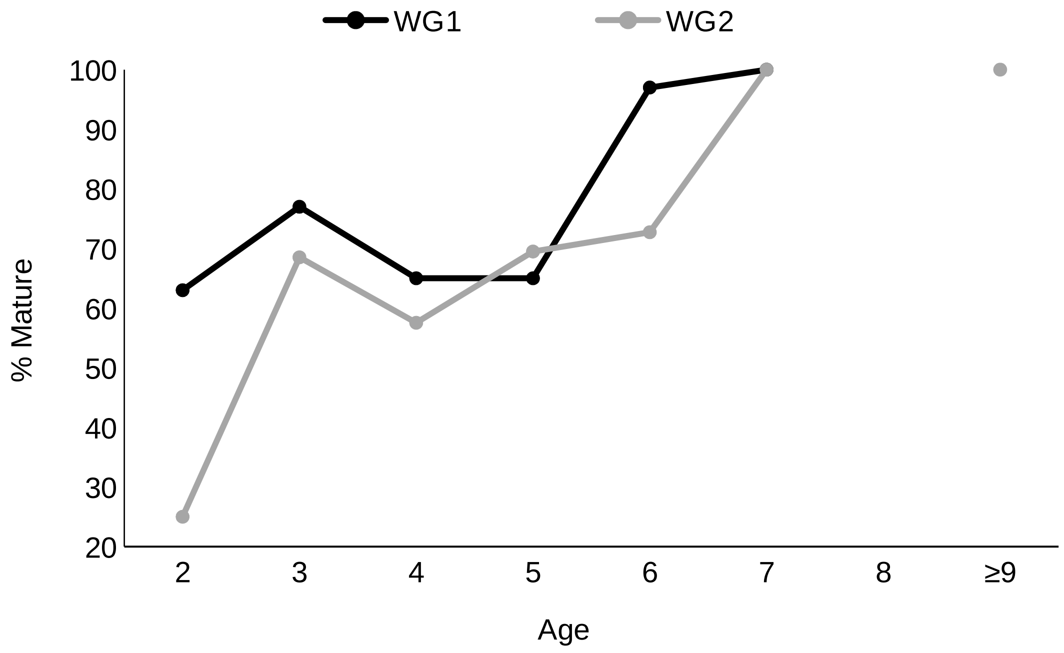

WG1
WG2
100
90
80
70
60
50
40
30
20
% Mature
2
3
4
5
6
7
8
≥9
Age
