# Supplementary material for: Temporal and spatial comparisons of the reproductive biology of northern Gulf of Mexico (USA) red snapper (Lutjanus campechanus) collected a decade apart
Source: PLoS One. 2017 Mar 29;12(3):e0172360. doi: 10.1371/journal.pone.0172360 (PMC5371290; doi:10.1371/journal.pone.0172360)
Supplement: Supplementary file 20 [file pone.0172360.s023.docx]

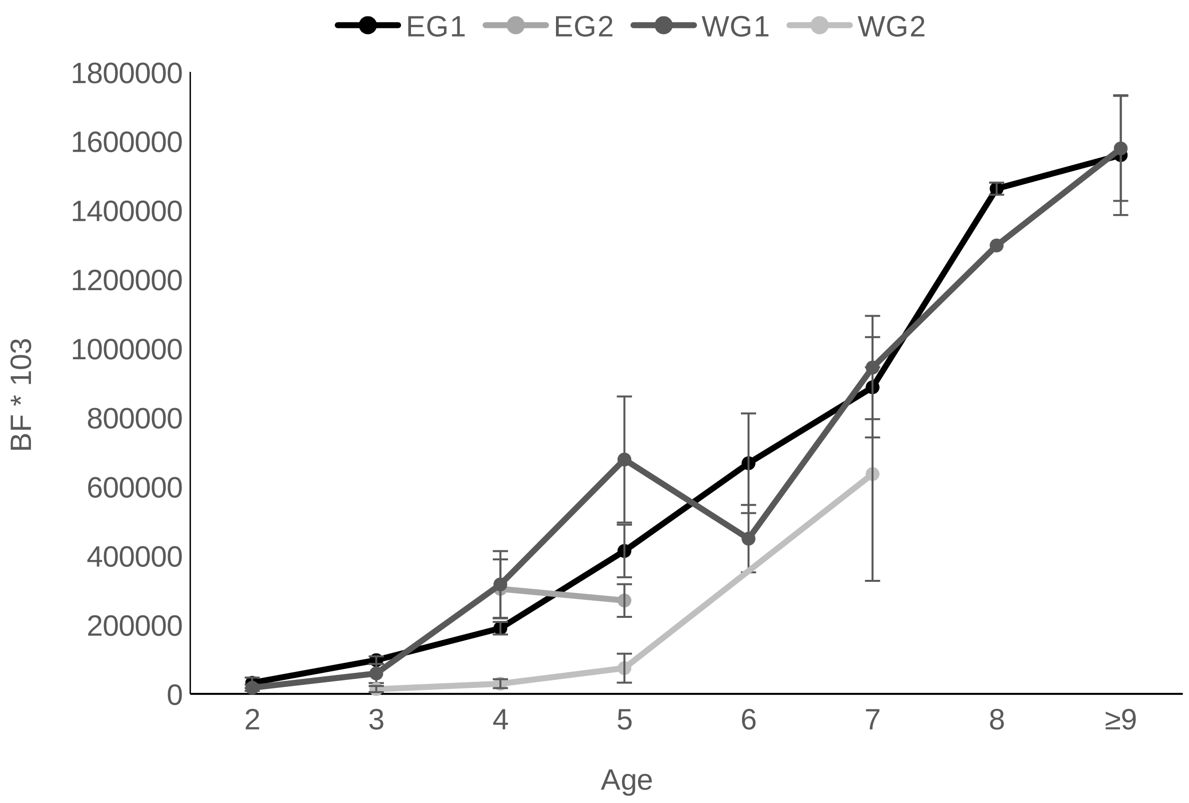

EG1
EG2
WG1
WG2
BF * 103
1800000
1600000
1400000
1200000
1000000
800000
600000
400000
200000
0
2
3
4
5
6
7
8
≥9
Age
